# Neurotrophins and Galectin-3: Hidden Keys in Neuroinflammation—A Narrative Review

**DOI:** 10.3390/ijms27093742

**Published:** 2026-04-23

**Authors:** Bojana Simovic Markovic, Irfan Corovic, Marina Mitrovic, Nemanja Jovicic, Dragica Selakovic, Miodrag Sreckovic, Gvozden Rosic

**Affiliations:** 1 Center for Molecular Medicine and Stem Cell Research, Faculty of Medical Sciences, University of Kragujevac, 34000 Kragujevac, Serbia; bojana.simovic@gmail.com (B.S.M.); ira.corovic@gmail.com (I.C.); mitrovicmarina34@gmail.com (M.M.); 2 Department of Medical Biochemistry, Faculty of Medical Sciences, University of Kragujevac, 34000 Kragujevac, Serbia; 3 Department of Histology and Embryology, Faculty of Medical Sciences, University of Kragujevac, 34000 Kragujevac, Serbia; nemanjajovicic.kg@gmail.com; 4 Department of Physiology, Faculty of Medical Sciences, University of Kragujevac, 34000 Kragujevac, Serbia; grosic@fmn.kg.ac.rs; 5 Department of Internal Medicine, Faculty of Medical Sciences, University of Kragujevac, 34000 Kragujevac, Serbia; sreckovic7@gmail.com; 6 Clinic of Cardiology, University Clinical Center Kragujevac, 34000 Kragujevac, Serbia

**Keywords:** neurotrophins, Galectin-3, BDNF, neuroinflammation, microglia, neurodegenerative diseases, psychiatric disorders

## Abstract

Galectin-3 (Gal-3) is a multifunctional molecule that exerts pleiotropic effects in inflammatory responses and contributes to the pathogenesis of numerous immune-mediated diseases. Although Gal-3 has been known for more than five decades, it remains a lectin with intriguing and not yet fully elucidated properties. The existing body of evidence underscores the importance of Gal-3 in the regulation of homeostatic and inflammatory processes. Neurotrophins are traditionally recognized as key regulators of neuronal development, survival, and synaptic plasticity; nevertheless, accumulating evidence indicates that they also play important roles in immune regulation and neuroimmune communication. Importantly, neurotrophins are also produced by immune cells, including monocytes, macrophages, lymphocytes, and basophils, which express functional neurotrophin receptors including tropomyosin receptor kinase A (TrkA), tropomyosin receptor kinase A (TrkB), and p75 neurotrophin receptor (p75NTR). In this narrative review, we synthesize current evidence on neuroinflammation, neurotrophins, and Gal-3, with a particular focus on the molecular mechanisms involved in the crosstalk between neurotrophins and Gal-3 or immune cells. We further examine how this neuroimmune–neurotrophic crosstalk contributes to the pathogenesis of psychiatric and neurodegenerative disorders, as well as other neurological conditions. Finally, we discuss the emerging therapeutic potential of targeting neurotrophins and Gal-3 as modulators of neuroinflammation.

## 1. Introduction

Neuroinflammation represents a fundamental response of the central nervous system (CNS) to injury, infection, or disease and is primarily mediated by the activation of resident immune cells such as microglia and astrocytes. While acute inflammatory responses may exert protective effects, persistent or dysregulated inflammation promotes neuronal injury and contributes to the pathogenesis of numerous neurological and psychiatric disorders, including Alzheimer’s disease (AD), Parkinson’s disease (PD), multiple sclerosis (MS), depression, and cognitive dysfunction [1]. These processes are driven by the production of cytokines, chemokines, and reactive oxygen species released by glial cells, endothelial cells, and infiltrating immune cells. Among these components, microglia play a central role as innate immune sentinels of the CNS; however, sustained microglial activation can amplify inflammatory signaling and promote neurodegeneration and neurobehavioral disturbances [2,3].

Galectins are β-galactoside-binding lectins involved in diverse biological processes, including cell communication, apoptosis, and the regulation of immune-related functions [4,5,6,7,8,9,10,11,12]. Among them, galectin-3 (Gal-3) is widely expressed by immune and inflammatory cells such as macrophages, dendritic cells, mast cells, natural killer cells, and activated T and B lymphocytes, where it regulates both innate and adaptive immune responses [13]. Through intra- and extracellular interactions with glycans, Gal-3 can influence immune cell activation, migration, and inflammatory signaling, positioning it as a potential mediator of immune-driven pathology.

At the same time, neurotrophic signaling plays a key role in supporting neuronal viability, synaptic function, and adaptive responses to injury. Neurotrophins, including nerve growth factor (NGF), brain-derived neurotrophic factor (BDNF), neurotrophin-3 (NT-3), and neurotrophin-4/5 (NT-4/5), regulate neuronal and glial survival, as well as axonal and dendritic growth, synaptogenesis, and synaptic remodeling during development and throughout adulthood [14]. Growing evidence indicates that neurotrophins are involved in the bidirectional interactions between the nervous and immune systems in both physiological and pathological states.

Although both Gal-3 and neurotrophins have been extensively studied individually, their potential interaction within neuroinflammatory processes remains insufficiently explored. This represents an important gap in the current understanding of neuroimmune regulation. This review addresses the interplay between neurotrophins and Gal-3 in neuroinflammation and evaluates its relevance to the pathogenesis of major neurological and psychiatric conditions, with an emphasis on therapeutic implications.

## 2. Neurotrophins

### 2.1. Neurotrophins in Immune Response

Neurotrophins are traditionally recognized as key regulators of neuronal development, survival, and synaptic plasticity; nevertheless, accumulating evidence indicates that they also play important roles in immune regulation and neuroimmune communication. In addition to neurons, neurotrophins are produced by immune cells, including monocytes, macrophages, lymphocytes, and basophils, which express functional receptors for functional neurotrophin receptors such as TrkA, TrkB, and p75NTR [15,16]. Through receptor-dependent signaling, neurotrophins mediate bidirectional communication between the nervous and immune systems, influencing immune cell survival, differentiation, activation, and effector functions. BDNF has been implicated in chronic inflammatory conditions such as inflammatory bowel disease and rheumatoid arthritis, where altered BDNF availability may affect immune cell polarization, nociceptive processing, and neuropsychiatric comorbidities associated with persistent inflammation [15]. Similarly, immune cells within the inflamed tissues can locally produce BDNF, supporting neuronal resilience and contributing to the concept of “neuroprotective immunity” [16]. Moreover, NGF-TrkA signaling contributes to thymic development and thymocyte survival, indicating a role for neurotrophins in immune homeostasis and central tolerance [17]. The expression of NGF can also be induced by pro-inflammatory cytokines such as interleukin-1 and tumor necrosis factor-α, forming feedback loops that link immune activation with neurotrophin signaling. Furthermore, elevated NGF levels observed in autoimmune disorders such as systemic lupus erythematosus further support the involvement of neurotrophin pathways in immunopathology, although the biological consequences likely depend on inflammatory intensity, receptor balance, and disease stage [18]. Within barrier tissues, including the skin and airways, neurotrophins mediate communication between immune cells and sensory nerve fibers, thereby contributing to inflammatory responses in conditions such as asthma and atopic dermatitis [19]. In addition, neurotrophins participate in immune tolerance during pregnancy by regulating neuroendocrine–immune interactions at the maternal–placental interface [20]. Rather than acting as simple growth factors, neurotrophins function as context-dependent regulators that shape immune activation, resolution, and tissue adaptation. While these mechanisms are evident in peripheral immunity, a parallel, yet functionally more consequential, neurotrophin–immune interface operates within the CNS.

### 2.2. Neurotrophins in Neuroimmune Regulation—Overview

Within the CNS, neurotrophins represent key mediators of neuroimmune communication, operating in an environment where immune signaling directly influences synaptic integrity and neuronal survival. Unlike peripheral tissues, neuroinflammatory processes in the CNS occur in close proximity to neurons and synapses, thereby amplifying the functional consequences of immune–neurotrophin interactions. NGF and BDNF are produced not only by neurons but also by astrocytes, microglia, mast cells, and infiltrating lymphocytes, all of which express neurotrophin receptors and integrate neurotrophin signaling into inflammatory and stress-response pathways [16,18,21,22,23]. Through receptor-specific mechanisms, neurotrophins regulate glial activation, cytokine production, and neuronal survival programs. In many contexts, NGF and BDNF promote anti-inflammatory or reparative glial phenotypes, enhance neuronal resilience, and stabilize synaptic function. Furthermore, astrocytic exposure to pro-inflammatory cytokines such as TNF-α can upregulate NGF and BDNF expression through NF-κB-dependent mechanisms, suggesting that neurotrophin induction may act as an endogenous compensatory response to acute inflammatory injury [16,22].

However, the effects of neurotrophin signaling during neuroinflammation are strongly context-dependent. Although Trk receptor activation generally promotes pro-survival signaling, excessive or dysregulated signaling through p75NTR may trigger apoptotic cascades, synaptic destabilization, and ultimately neuronal loss [21,23,24]. Consequently, the biological outcome depends on receptor balance, inflammatory intensity, and the temporal dynamics of signaling. Furthermore, neurotrophin pathways are closely interconnected with cytokine networks, including IL-1β, TNF-α, and IL-6, forming bidirectional feedback loops in which cytokines regulate neurotrophin expression. In contrast, neurotrophins modulate immune signaling and glial activity [18,23]. Cytokines such as TNF-α and IL-6 can induce neurotrophin expression in astrocytes and neurons by activating the NF-κB and cAMP response element-binding protein (CREB) signaling pathways. In contrast, neuronal activity and cAMP signaling may further potentiate this response [25,26]. Conversely, neurotrophins can influence inflammatory signaling by regulating immune mediators and activating transcriptional programs linked to inflammatory and nociceptive responses [26]. In the context of neuroinflammatory and demyelinating conditions, neurotrophins may promote remyelination, facilitate oligodendrocyte precursor recruitment, and support anti-inflammatory glial phenotypes [16,27]. Conversely, reduced neurotrophin availability or altered neurotrophin-receptor signaling has been associated with impaired synaptic plasticity, enhanced neurotoxicity, and the progression of chronic neurodegenerative and psychiatric disorders [27,28]. The concept of “neuroprotective immunity” has emerged from observations that immune cells within CNS lesions produce neurotrophins such as BDNF, which support neuronal survival and tissue repair [16,27]. Nevertheless, persistent or dysregulated neuroimmune interactions may contribute to chronic inflammation and progressive neurodegeneration, emphasizing the importance of receptor specificity and cellular context in determining biological outcomes [16,24,28].

Collectively, neurotrophins play a central and context-dependent role in neuroimmune regulation. By integrating inflammatory signals with neuronal survival and repair pathways, neurotrophin signaling critically influences the balance between neuroprotection and neurodegeneration in neuroinflammatory disorders.

## 3. Galectin Family: Outstanding Gal-3 Molecule

### 3.1. Overview

Galectins are β-galactoside-binding lectins that play key roles in regulating cell growth, adhesion, migration, immune responses, and tumor progression [29]. The term galectin was introduced by Barondes and colleagues in 1994 to describe lectins that possess a strong affinity for β-galactosides and conserved carbohydrate-recognition domains (CRDs) [29]. Galectins are evolutionarily conserved proteins sharing a CRD of approximately 130 amino acids, which is responsible for β-galactoside binding [30]. To date, 17 mammalian galectins have been identified. They are structurally classified into three groups: prototype galectins containing a single CRD (e.g., Gal-1), tandem-repeat galectins with two CRDs connected by a linker (e.g., Gal-8 and Gal-9), and chimera-type galectins, represented solely by Gal-3, which contains a single CRD linked to a non-lectin N-terminal domain [30]. Gal-3 is widely distributed across intracellular and extracellular compartments and can localize to the cytoplasm, nucleus, cell surface, or extracellular space depending on the cellular context [31]. Through interactions with multiple intra- and extracellular ligands, Gal-3 participates in diverse cellular processes, including pre-mRNA splicing, cell cycle regulation, apoptosis, and cell growth [32,33,34,35]. Extracellularly, Gal-3 can bind to glycoconjugates on the cell surface and within the extracellular matrix, thereby mediating cell–cell and cell–matrix interactions [32,33]. Although it is monomeric in solution, Gal-3 oligomerizes into pentamers upon binding multivalent carbohydrates, enabling the cross-linking of glycoproteins and the formation of lattice-like structures that regulate receptor signaling and immune cell migration [32,33,36].

### 3.2. Deciphering the Gal-3 Role in Immune Response

Gal-3 is a multifunctional lectin that plays a pleiotropic role in mediating both acute and chronic inflammatory responses, and it contributes to the pathogenesis of numerous immune-mediated diseases. A growing body of evidence indicates that Gal-3 is an important regulator of innate immunity. It functions as a chemoattractant for monocytes and macrophages, and Gal-3-deficient mice exhibit reduced inflammatory responses with decreased leukocyte infiltration, whereas administration of recombinant Gal-3 enhances monocyte recruitment and exacerbates inflammation [32,37]. Gal-3 also participates in macrophage activation by interacting with the CD98 receptor and activating the PI3K signaling pathway, thereby promoting alternative macrophage polarization [38]. In addition, Gal-3 acts as an opsonin, facilitating macrophage clearance of apoptotic neutrophils, whereas Gal-3-deficient macrophages exhibit impaired phagocytic capacity and delayed engulfment of target cells [39,40]. Experimental studies further demonstrate that Gal-3 can promote activation of the NLRP3 inflammasome and IL-1β production in macrophages, highlighting its role in amplifying inflammatory responses [9].

Gal-3 also regulates the function of other immune cell populations. It contributes to dendritic cell migration and cytokine production, as dendritic cells derived from Gal-3-deficient mice show impaired chemokine-driven migration and reduced trafficking to lymph nodes [41,42,43,44]. In adaptive immunity, Gal-3 expression is induced during T-cell activation and differentiation, and it is detected in activated, memory, and regulatory T cells [31,45]. Notably, the functional consequences of Gal-3 depend on its cellular localization. Extracellular Gal-3 can induce apoptosis in activated T cells by binding to surface receptors such as CD45, CD71, CD7, and CD29, thereby triggering mitochondrial apoptotic pathways involving cytochrome c release and caspase-3 activation [31,46,47]. In contrast, intracellular Gal-3 promotes cell survival, inhibits apoptosis, and modulates T-cell receptor signaling [31].

In summary, Gal-3 acts as a context-dependent regulator of inflammatory responses. It can enhance inflammation by promoting immune cell recruitment, cytokine production, and inflammasome activation, while intracellular Gal-3 may sustain inflammatory responses by supporting the survival of activated immune cells. Through these mechanisms, Gal-3 functions as a key modulator of both innate and adaptive immunity, contributing to the persistence and amplification of inflammatory processes.

### 3.3. Pivotal Role of Gal-3 in Neuroinflammation

**Figure 1:**
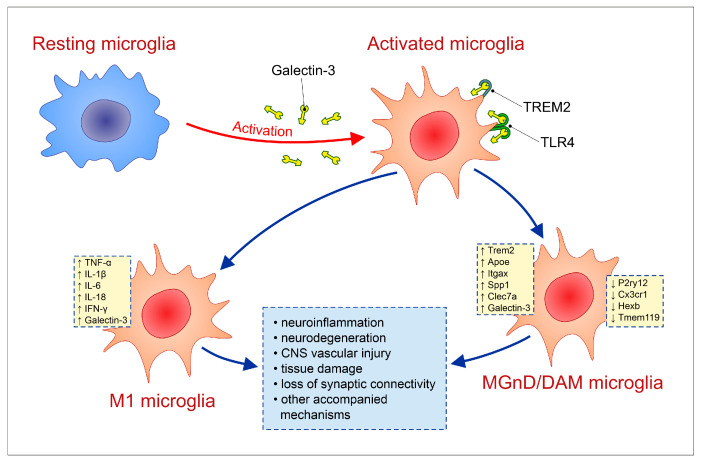
The mechanism of microglial activation by Gal-3.

Microglial activation represents a central mechanism linking Gal-3 to neuroinflammation (Figure 1). Gal-3 expression is strongly associated with activated microglia, whereas resting microglia do not typically express this lectin [48,49]. Recent studies have identified a common activated microglial phenotype observed across several neurodegenerative diseases, including Alzheimer’s disease, Parkinson’s disease, and multiple sclerosis. This phenotype has been described as disease-associated microglia (DAM) or microglia with a neurodegenerative phenotype (MGnD) [48,49]. Both of these states are characterized by the downregulation of homeostatic microglial genes such as *P2ry12*, *Cx3cr1*, *Hexb*, and *Tmem119*, together with the upregulation of genes associated with inflammatory activation, including *Trem2*, *Apoe*, *Itgax*, *Spp1*, and *Clec7a* [48,49]. Although Gal-3 was not initially reported as being altered in DAM, it was markedly upregulated in the MGnD phenotype [49]. Subsequent studies have further demonstrated that Gal-3 is one of the most strongly upregulated genes in microglia during brain disease, supporting its role as a key mediator of neuroinflammatory activation [50,51,52].

## 4. Psychiatric Disorders

### 4.1. Schizophrenia

Schizophrenia is a severe psychiatric disorder affecting approximately 0.3% of the global population, characterized by disturbances in cognition, perception, behavior, and emotional regulation [53,54]. Cognitive impairment represents a core feature of the disorder, affecting memory, attention, processing speed, and executive functioning. Although the pathophysiology of schizophrenia is multifaceted, increasing evidence indicates that disruptions in neurotrophic signaling and immune–inflammatory processes play a key role in the disease’s underlying mechanisms. BDNF is a central regulator of neuronal development, synaptic plasticity, and neuroprotection. Numerous studies have reported reduced BDNF levels in both the central nervous system and peripheral circulation of patients with schizophrenia, with lower BDNF levels being consistently associated with cognitive deficits, particularly in memory, visual learning, processing speed, and reasoning [55,56,57,58,59]. These peripheral BDNF levels serve as a reliable proxy for BDNF activity in the CNS [60], as the protein’s capacity to cross the blood–brain barrier (BBB) ensures that systemic levels accurately reflect neurobiological conditions [61]. BDNF primarily acts through activation of the TrkB receptor, which regulates intracellular signaling pathways such as PI3K/AKT, PLCγ, and MAPK/ERK, all of which are essential for neuronal survival and synaptic remodeling [59]. Consequently, disruption of these pathways may contribute to the neurodevelopmental abnormalities and synaptic dysfunction observed in schizophrenia. BDNF signaling also interacts with neurotransmitter systems implicated in the disorder, including dopamine, glutamate, serotonin, and GABA, and reduced BDNF availability may contribute to the imbalance between excitatory and inhibitory neuronal activity [58,62,63]. Genetic and epigenetic mechanisms, including alterations in synaptic genes and epigenetic regulation of the BDNF gene, may further influence BDNF expression and clinical variability in schizophrenia [62]. Although antipsychotic treatment can increase peripheral BDNF levels, these effects appear heterogeneous and depend on treatment type and patient characteristics [55,58,62]. In addition to neurotrophic alterations, immune–inflammatory dysregulation is frequently observed in schizophrenia. Elevated levels of cytokines such as IL-2, IL-6, IL-8, and TNF-α have been repeatedly reported in patients [28,64,65,66,67]. Importantly, inflammatory signaling and BDNF pathways are closely interconnected. Cytokines can modulate neurotrophic signaling through pathways involving STAT3 and TRAF6, influencing kinase activity, immune regulation, and neuronal plasticity. Consequently, reduced BDNF signaling may exacerbate inflammatory responses, while chronic inflammation can further suppress neurotrophic support, creating a self-reinforcing cycle that contributes to synaptic dysfunction and neurotoxicity in schizophrenia [28,68,69].

Clinical and translational studies consistently support the interaction between neurotrophic and inflammatory mechanisms in schizophrenia (Table 1). Specifically, reduced BDNF levels, together with elevated inflammatory mediators, have been associated with impairments in executive function, working memory, and attention, suggesting that both diminished neurotrophic support and persistent immune activation may jointly disrupt the neural circuits that support higher cognitive processes [64,65]. In chronic schizophrenia, these alterations appear to be particularly pronounced. Indeed, case–control studies involving chronically medicated or long-term hospitalized patients have demonstrated reduced BDNF levels accompanied by increased cytokines such as IL-2, IL-6, and IL-8, with BDNF–cytokine interactions significantly predicting executive dysfunction and cognitive impairment [62,64,65]. Meta-analytic evidence further supports these observed relationships. A systematic review including 21 studies and 2449 individuals diagnosed with schizophrenia-spectrum disorders reported that higher peripheral BDNF levels were modestly associated with better cognitive performance (r = 0.12). In contrast, elevated C-reactive protein (CRP) levels were associated with decreased cognitive function (r = −0.13). BDNF levels were particularly associated with performance on verbal memory, working memory, processing speed, and verbal fluency, suggesting that reduced neurotrophic signaling, together with systemic inflammation, contributes to cognitive dysfunction in schizophrenia [70]. Importantly, similar neuroimmune alterations appear early in the disease course. In a cohort of 84 patients with first-episode schizophrenia and 80 control subjects, elevated IL-1β and IL-6 together with reduced BDNF levels were associated with deficits in attention, working memory, and executive functioning, indicating that inflammatory activation and diminished neurotrophic support may contribute to cognitive impairment from the early stages of the illness [71]. Additional studies suggest that altered processing of neurotrophic factors may further contribute to this pathology. The dysregulation of the MMP-9/proBDNF/BDNF signaling axis has been reported in schizophrenia, indicating impaired conversion of proBDNF to its mature form and disruption of synaptic remodeling [72]. Interactions between inflammatory and apoptotic pathways have also been observed. For example, higher IL-1β levels during relapse were associated with more severe negative symptoms, while Fas ligand (FasL) levels were negatively related to treatment response [73]. Biomarker studies also indicate substantial heterogeneity in inflammatory responses among patients. In a cross-sectional study of 54 patients with schizophrenia and 40 controls, multiple inflammatory mediators, such as IL-1β, IL-2, IL-4, IL-6, B-cell activating factor (BAFF), IFN-α, and granulocyte-macrophage colony-stimulating factor (GM-CSF) and growth factors, such as neuregulin-1 beta 1 (NRG1-β1), glial cell line-derived neurotrophic factor (GDNF) were elevated, whereas TNF-α and NGF-β were reduced, with cluster analyses identifying subgroups characterized by distinct inflammatory profiles [74]. Metabolic factors may further interact with this network. In a study of 301 patients with chronic schizophrenia, leptin levels were positively associated with BDNF and inflammatory cytokines, including IL-6 and IL-17A, while negatively correlated with overall psychopathology severity, supporting an integrated neuroimmune–metabolic framework [75]. Treatment-related factors may also influence these biological interactions. A case–control study including 150 patients with schizophrenia and 162 controls demonstrated elevated IL-1β, IL-2, and IL-6 levels together with reduced BDNF concentrations, with these alterations associated with deficits in attention, executive function, and visuospatial working memory; notably, cognitive impairment was more pronounced among patients treated with typical antipsychotics [76]. Interventional studies similarly suggest that therapeutic modulation of inflammation may influence neurotrophic signaling, as clinical improvement during electroconvulsive therapy (ECT) was accompanied by reductions in IL-6 and increases in BDNF levels [77]. Experimental studies further support causal links between inflammatory signaling and neurotrophic dysfunction. In a ketamine-induced mouse model of schizophrenia, pharmacological suppression of inflammatory pathways (TNF-α, IL-6, NF-κB, and MAPK) was accompanied by increased BDNF expression and improved behavioral outcomes [78]. In addition, post-mortem analyses also revealed reduced expression of BDNF, NURR1, and full-length TrkB, together with increased expression of truncated TrkB and p75 receptors, particularly in brains with strong inflammatory signatures, suggesting that neuroinflammation may shift BDNF signaling toward reduced trophic support and increased apoptotic signaling in dopaminergic neurons [68]. Overall, accumulating clinical, experimental, and post-mortem evidence collectively indicates that neuroinflammatory activation interacts with impaired BDNF signaling to disrupt synaptic plasticity and cognitive functioning in schizophrenia.

In addition to cytokines and neurotrophic factors, lectin family proteins, such as Gal-3, may also contribute to immune signaling pathways implicated in schizophrenia [79,80,81,82]. Galectins are β-galactoside-binding lectins that are involved in immune regulation, cell adhesion, and inflammatory signaling [83], and emerging evidence suggests that Gal-3 may serve as a peripheral marker of neuroimmune activity in psychotic disorders. Some clinical studies indicate that Gal-3 levels vary across disease stages. In a study including 77 patients with first-episode psychosis, 45 patients with relapse, 27 patients in remission, and 18 controls, serum Gal-3 levels were reduced during the acute phases of illness but significantly elevated during remission. These changes were accompanied by increased IL-33 and soluble ST2 levels, suggesting that Gal-3 and the IL-33/ST2 pathway may participate in stage-dependent inflammatory signaling in schizophrenia [79]. Additional studies further support links between Gal-3 and immune activation. In patients with schizophrenia in remission, Gal-3 levels correlated positively with TNF-α, IL-23, and soluble ST2, whereas associations with illness duration and reduced TGF-β suggested a role in systemic inflammatory regulation during stabilized disease stages [80]. Similarly, another case–control study demonstrated reduced circulating Gal-3 levels in patients with schizophrenia, with higher Gal-3 concentrations associated with greater severity of negative symptoms [81]. Experimental data also support the involvement of Gal-3 in schizophrenia-related neuroinflammation. In a cuprizone-induced mouse model, treatment with syringic acid improved behavioral deficits and increased markers of neuronal and myelin integrity (MBP, NRG-1, and PI3K/Akt signaling), while reducing Gal-3, TNF-α, GFAP, and GSK-3β, indicating attenuation of neuroinflammatory and glial activation processes [82].

Together, these findings suggest that Gal-3 participates in inflammatory pathways that intersect with mechanisms regulating neuroplasticity and neuronal survival. Since neuroinflammatory signaling can influence neurotrophic pathways, particularly BDNF-dependent mechanisms, Gal-3 may represent an additional component of the broader neuroimmune–neurotrophic network implicated in schizophrenia.

### 4.2. Major Depressive Disorder

Major depressive disorder (MDD) is a highly prevalent psychiatric disorder characterized by persistent low mood, anhedonia, cognitive dysfunction, and somatic symptoms that markedly impair quality of life [84]. Increasing evidence indicates that disruptions in neurotrophic signaling and immune–inflammatory processes contribute to its pathophysiology (Table 1). According to the neurotrophic hypothesis of depression, reduced BDNF signaling contributes to neuronal atrophy and impaired synaptic connectivity, whereas restoration of BDNF levels is associated with symptom improvement [85,86,87,88,89,90]. Both clinical and experimental studies consistently report reduced peripheral and central BDNF levels in patients with MDD, with circulating BDNF inversely correlated with symptom severity [88,89,90]. Chronic stress, a major risk factor for depression, suppresses BDNF expression in the hippocampus and prefrontal cortex, leading to dendritic atrophy and synaptic dysfunction, which may be partially reversed by antidepressant treatment [86,91,92]. The balance between mBDNF and its precursor, proBDNF, is also critical, as proBDNF may promote depressive-like behaviors, whereas mBDNF supports neuronal resilience [87,92]. Antidepressant treatments, including rapid-acting agents such as ketamine, increase BDNF signaling by activating the TrkB receptor and downstream pathways that regulate synaptic plasticity [85,86,87,88,89,90]. In addition to BDNF, other neurotrophins may contribute to depression-related neurobiology. The levels of NGF are found to be reduced in patients with MDD and inversely associated with disease severity, whereas NT-3 and NT-4 may also contribute to disease mechanisms [93]. Importantly, neurotrophic alterations are closely linked to immune–inflammatory activation, as pro-inflammatory cytokines, including IL-1β, IL-6, and TNF-α, can interfere with neurotrophic signaling and contribute to impaired neuroplasticity and the development of depressive symptoms [94,95,96].

Clinical studies increasingly indicate that MDD involves coordinated disturbances in neurotrophic and immune–inflammatory pathways. Consistent with this, clinical studies frequently report reduced circulating BDNF together with elevated pro-inflammatory cytokines, thereby supporting the concept that impaired neuroplasticity and immune activation are mechanistically linked components of depressive pathophysiology. For example, in a study including 30 patients with MDD and 30 healthy controls, serum BDNF levels were reduced, whereas IL-1β levels were increased. Furthermore, IL-1β positively correlated with depression severity, and BDNF showed an inverse association with symptom scores [97]. Further insight comes from studies examining different molecular forms of BDNF. Yoshida et al. [98] reported reduced serum mature BDNF (mBDNF) in 69 patients with MDD compared with 78 controls, while proBDNF and MMP-9 levels remained unchanged, suggesting that impaired processing of proBDNF into its biologically active form may contribute to neurotrophic dysfunction. Consistent with this mechanism, Yang et al. [99] demonstrated increased expression of proBDNF, p75NTR, and sortilin in peripheral blood mononuclear cells, accompanied by elevated levels of cytokines, including IL-1β, IL-6, and TNF-α. Functional experiments further showed that proBDNF stimulation increased cytokine release, whereas inhibition of p75NTR reduced inflammatory signaling, indicating that proBDNF–p75NTR signaling may act upstream of immune activation in depression. Longitudinal and biomarker studies further support the functional coupling between inflammatory and neurotrophic systems. In a cohort of 99 patients with severe late-life depression undergoing electroconvulsive therapy, the TNF-α/BDNF ratio was associated with depression severity, suggesting that the balance between inflammatory and neurotrophic signaling may more accurately represent disease activity than individual biomarkers [100]. Similarly, the combined measurement of BDNF and IL-6 identified the BDNF/IL-6 ratio as a potentially useful biomarker for differentiating MDD patients from healthy controls and predicting treatment response [101]. Neurotrophic dysregulation in depression may also extend beyond BDNF. In a study including 30 patients with MDD and 20 controls, NGF levels were significantly reduced, whereas indices of inflammatory neurotoxicity, including IL-1β, IL-6, TNF-α, IL-2, IFN-γ, and IL-17, were increased. Importantly, a reduced NGF/neurotoxicity ratio was associated with greater depression severity, anxiety, suicidal behavior, and illness recurrence, suggesting that diminished neurotrophic protection relative to inflammatory neurotoxicity may represent a key mechanism contributing to depressive pathology [102].

Experimental studies provide important mechanistic insights into the bidirectional interaction between inflammatory signaling and neurotrophic pathways in depression. Animal models consistently demonstrate that inflammatory activation disrupts BDNF-dependent synaptic plasticity and neurogenesis, contributing to depressive-like behaviors. For instance, in a chronic mild stress (CMS) rat model, depressive-like behaviors were accompanied by increased expression of pro-inflammatory cytokines (IL-1β, TNF-α, IL-6, and IL-18) and reduced anti-inflammatory mediators (IL-10, TGF-β), together with decreased BDNF mRNA expression in the hippocampus and hypothalamus, indicating that cytokine imbalance can suppress neurotrophic signaling and neuronal regeneration [103]. Subsequent studies confirmed that inflammatory activation directly interferes with BDNF-TrkB synaptic signaling. In an LPS-induced mouse model, neuroinflammation led to dendritic spine loss and synaptic protein dysregulation, which was associated with impaired BDNF signaling. Pharmacological inhibition of the MNK1/2-eIF4E pathway restored synaptic protein expression, normalized BDNF activity, and attenuated depressive-like behaviors; these effects were reversed by TrkB blockade, thus confirming the central role of BDNF signaling in inflammation-induced behavioral changes [104]. Upstream inflammatory mechanisms have also been implicated in these phenomena. Specifically, the activation of the NLRP1 inflammasome in stress models increased IL-1β, IL-18, IL-6, and TNF-α while reducing hippocampal BDNF expression. Genetic knockdown of Nlrp1a attenuated inflammation, restored BDNF levels, and improved depressive-like behaviors, indicating that inflammasome signaling can impair neurotrophic pathways through CXCL1/CXCR2-BDNF mechanisms [105]. Additional evidence suggests that BDNF also modulates neuroimmune responses. In BDNF heterozygous mice exposed to LPS, reduced BDNF expression amplified neuroinflammatory responses in a sex-dependent manner, particularly in females, thus highlighting a role for BDNF in regulating susceptibility to inflammation-induced neural dysfunction [106]. Finally, models of comorbid chronic pain and depression demonstrate that glial dysfunction may further contribute to neuroimmune–neurotrophic interactions. Chronic stress combined with experimental colitis induced NF-κB-mediated inflammation, reduced microglial density in the medial prefrontal cortex, and decreased BDNF and CREB expression, linking glial activation to impaired synaptic plasticity in inflammatory depression [107]. Collectively, experimental evidence indicates that inflammatory activation disrupts BDNF signaling through cytokine-mediated suppression, inflammasome activation, and glial dysfunction, thereby linking immune activation to impaired neurogenesis, synaptic plasticity, and depressive-like behaviors.

Moreover, Gal-3 has emerged as a potential link between neuroinflammation and neuroplasticity disturbances in major depressive disorder. Population-based data derived from the Dallas Heart Study (*n* = 2554) demonstrated that circulating Gal-3 levels significantly predicted depressive symptom severity (β = 0.055, *p* = 0.015) [108]. Similarly, in a cohort of 283 patients with type 1 diabetes, those individuals with depression exhibited higher Gal-3 levels than non-depressed patients, and elevated Gal-3 was independently associated with depression (adjusted OR = 3.5) [109]. Experimental evidence further supports a mechanistic role for Gal-3 in mood regulation. Specifically, Gal-3 knockout mice exhibited increased depressive-like behavior in behavioral tests together with reduced BDNF levels in the prefrontal cortex, indicating that Gal-3 may influence neurotrophic signaling [110]. Collectively, these findings suggest that Gal-3 may represent a molecular interface, linking immune activation, neuroinflammation, and impaired BDNF-dependent neuroplasticity in depression, although the available data remains limited.

### 4.3. Anxiety

Anxiety disorders, including phobic disorders, panic disorder, and generalized anxiety disorder, are the most common mental disorders in Europe, affecting approximately 14% of the population [111]. Increasing evidence indicates that neurotrophic and immune–inflammatory mechanisms contribute to their pathophysiology (Table 1). Neurotrophins play a significant role in the development and regulation of fear and anxiety circuits, particularly within limbic regions such as the amygdala and hippocampus. Alterations in BDNF expression during critical developmental periods may produce long-lasting effects on fear learning and anxiety-related behaviors [112,113]. At the circuit level, BDNF modulates serotonergic signaling in the basolateral amygdala, and disruption of BDNF signaling reduces serotonin availability and alters GABAergic and glutamatergic transmission, leading to increased neuronal excitability and anxiety-like behavior [114]. Similarly, clinical studies have also reported reduced circulating BDNF levels in individuals with anxiety disorders, with BDNF dynamics associated with treatment response in conditions such as panic disorder [115,116]. Parallel to neurotrophic alterations, anxiety disorders are also associated with immune–inflammatory dysregulation, characterized by decreased anti-inflammatory IL-10 and increased pro-inflammatory cytokines, such as TNF-α and IFN-γ, resulting in elevated pro- to anti-inflammatory cytokine ratios [117]. Chronic stress represents a major environmental factor linking these processes by activating microglia–astrocyte inflammatory cascade, which subsequently suppresses BDNF expression and disrupts synaptic plasticity in anxiety-related circuits [118]. Although direct mechanistic evidence linking inflammation and neurotrophic signaling in anxiety remains limited, emerging experimental studies suggest that inflammatory pathways can directly regulate BDNF expression. In an LPS-induced anxiety model, IL-33 was markedly upregulated in the basolateral amygdala. Genetic deletion of IL-33 prevented anxiety-like behavior, whereas astrocyte-specific IL-33 overexpression induced anxiogenic responses. Mechanistically, IL-33 suppressed BDNF expression via NF-κB signaling, leading to impaired GABAergic transmission in the amygdala. Pharmacological inhibition of NF-κB restored BDNF signaling and reversed anxiety-like behavior, identifying IL-33 as a potential molecular link between neuroinflammation and anxiety-related neural circuitry [119].

Additional mechanistic insight into the interaction between neuroinflammatory mediators and neurotrophic signaling in anxiety has been provided by experimental studies examining the role of Gal-3. In an experimental mouse model performed by Stajic and coworkers [120], genetic deletion of Gal-3 produced a complex behavioral phenotype characterized by increased anxiety-like behavior observed under basal conditions, accompanied by reduced hippocampal BDNF expression and decreased expression of GABA_A_ receptor subunits (α2 and α5). These changes were associated with lower hippocampal levels of pro-inflammatory cytokines IL-6 and TNF-α, suggesting that physiological levels of inflammatory signaling may be necessary for maintaining normal neurotrophic support within anxiety-regulating circuits. In contrast, under conditions of LPS-induced neuroinflammation, Gal-3 deficiency exerted the opposite behavioral effect. Wild-type mice exposed to LPS displayed marked anxiogenic responses accompanied by increased hippocampal IL-6, TNF-α, and TLR4 expression together with a reduction in BDNF levels and GABA_A_ receptor expression. Importantly, Gal-3 knockout prevented the LPS-induced increase in IL-6 and attenuated the decline in hippocampal BDNF expression, thereby mitigating anxiety-like behavior. Mechanistically, these findings suggest that Gal-3 modulates anxiety through context-dependent regulation of the neuroimmune–neurotrophic axis. Under physiological conditions, Gal-3 appears to support hippocampal BDNF signaling and GABAergic transmission, thereby exerting an anxiolytic influence. Conversely, during inflammatory activation, Gal-3 may amplify neuroinflammatory signaling through the Gal-3/TLR4 pathway, leading to suppression of BDNF expression and disruption of inhibitory neurotransmission [120]. Collectively, these data indicate that galectin-3 may function as a bidirectional regulator of anxiety-related neural circuits, linking inflammatory cytokine signaling (particularly IL-6 and TNF-α) with BDNF-dependent regulation of hippocampal GABAergic transmission.

**Table 1:** Experimental and clinical studies linking neurotrophins, inflammation, and Gal-3 in psychiatric disorders.

Study Design	Sample/Model	Key Inflammatory Markers	Neurotrophin Levels	Gal-3 Levels	Main Findings
***Schizophrenia***					
Observational clinical study [64]	92 chronic SCZ/60 HC	↑ IL-2, ↑ IL-6, ↑ IL-8	↓ BDNF	-	Combined neurotrophic and inflammatory dysregulation was associated with poorer cognitive performance.
Observational clinical study [65]	112 chronic SCZ	↑ IL-2, ↑ IL-6, ↑ IL-8, ↓ TNF-α	↓ BDNF	-	Interactions between BDNF and inflammatory cytokines significantly predicted executive dysfunction.
Meta-analysis [70]	2449 SCZ-spectrum patients	CRP is associated with cognition	Higher BDNF is associated with better cognition	-	Higher CRP predicted worse cognition, while higher BDNF modestly predicted better cognitive performance across several domains.
Observational clinical study [71]	84 first-episode SCZ/80 HC	↑ IL-1β, ↑ IL-6	↓ BDNF	-	Elevated cytokines and reduced BDNF were associated with impairments in attention, working memory, and executive function in early-stage schizophrenia.
Observational clinical study [72]	105 SCZ/76 HC	↑ MMP-9	↓ BDNF, ↑ proBDNF	-	Dysregulation of the MMP-9/proBDNF/BDNF axis suggests impaired neurotrophin processing contributing to synaptic dysfunction.
Observational clinical study [73]	53 SCZ relapse/45 HC	↑ IL-1β	BDNF negatively correlated with inflammation	-	Higher IL-1β predicted worse symptoms and poorer treatment response; apoptotic signaling (FasL) may interact with neuroinflammation and neurotrophic pathways.
Observational clinical study [74]	54 SCZ/40 HC	↑ IL-1β, ↑ IL-2, ↑ IL-4, ↑ IL-6, ↑ BAFF, ↑ IFN-α	Growth factor alterations	-	Identified high- and low-inflammation subgroups, suggesting inflammatory heterogeneity among schizophrenia patients.
Observational clinical study [75]	301 chronic SCZ	IL-6, IL-17A associated with leptin	BDNF correlated with leptin		Suggests a neuroimmune–metabolic interaction influencing clinical severity.
Observational clinical study [76]	150 SCZ/162 HC	↑ IL-1β, ↑ IL-2, ↑ IL-6	↓ BDNF	-	Inflammatory activation and reduced BDNF were associated with cognitive deficits, particularly in patients receiving typical antipsychotics.
Prospective [77]	35 TRS patients	↓ IL-6 after treatment	↑ BDNF (trend)	-	Symptom improvement after ECT was associated with reduced inflammation and increased neuroplasticity markers.
Experimental [78]	Ketamine-induced schizophrenia mice	↓ TNF-α, ↓ IL-6, ↓ NF-κB	↑ brainBDNF expression	-	Anti-inflammatory treatment improved schizophrenia-like behaviors and restored neurotrophic signaling.
Post-mortem [68]	65 SCZ/64 controls	High inflammation subgroup (IL-6, IL-1β, TNF-α)	↓ brain BDNF, ↓ TrkB+, ↑ p75	-	Neuroinflammation shifts BDNF signaling toward reduced trophic support and increased apoptotic signaling in dopamine neurons.
Observational clinical study [79]	77 FEP, 45 SCZ relapse, 27 SCZ remission, 18 HC	↑ IL-33, ↑ sST2 during exacerbation	-	↓ in FEP and relapse; ↑ in remission	Gal-3 levels were lower in acute stages of schizophrenia but significantly elevated in remission, suggesting stage-dependent immune alterations.
Observational clinical study [80]	27 SCZ remission/18 HC	TNF-α, IL-23, sST2 correlated with Gal-3	-	Not significantly different (focus on correlations)	Gal-3 positively correlated with TNF-α, IL-23, and sST2 and inversely with leukocyte counts, indicating involvement in systemic inflammatory regulation.
Observational clinical study [81]	48 SCZ/44 HC	-	-	↓ in schizophrenia	Serum Gal-3 levels were significantly reduced in schizophrenia and positively correlated with negative symptom severity.
Experimental animal model [82]	Cuprizone-induced schizophrenia mice	↓ TNF-α, ↓ GFAP, ↓ GSK-3β	↑ NRG-1, ↑ MBP	↓ after treatment	Syringic acid improved behavioral abnormalities, reduced Gal-3 and inflammatory markers, and enhanced myelin-related proteins and PI3K/Akt signaling.
***Major Depressive Disorder***					
Observational clinical study [97]	30 MDD patients/30 healthy controls	↑ IL-1β	↓ BDNF	-	IL-1β positively correlated with depression severity, whereas BDNF showed a negative association with symptom severity.
Observational clinical study [98]	69 MDD patients + 78 healthy controls	MMP-9 measured	↓ mBDNF; proBDNF unchanged	-	Serum mBDNF was significantly reduced in MDD, whereas proBDNF and MMP-9 levels were unchanged. MMP-9 correlated with depression severity and functional outcomes.
Observational clinical study + in vitro experiments [99]	32 MDD patients + 20 healthy controls; PBMCs	↑ IL-1β, IL-6, TNF-α	↑ proBDNF; ↑ p75NTR; ↑ sortilin	-	Increased proBDNF/p75NTR signaling in immune cells, particularly CD4^+^ and CD8^+^ T lymphocytes. proBDNF stimulated cytokine production in PBMCs, while p75NTR blockade reduced cytokine release.
Longitudinal clinical study (ECT treatment) [100]	99 patients with severe late-life depression	IL-6, TNF-α	BDNF	-	Interaction between TNF-α and BDNF predicted depression severity during treatment. Higher TNF-α/BDNF ratio was associated with more severe depressive symptoms.
Observational clinical study [101]	MDD patients vs healthy controls	IL-6	BDNF	-	The serum BDNF/IL-6 ratio effectively differentiated MDD patients from controls and correlated with treatment response.
Observational clinical study [102]	30 MDD patients + 20 healthy controls	IL-1β, IL-6, TNF-α, IL-2, IFN-γ, IL-17	↓ NGF	-	NGF levels were significantly reduced in MDD, particularly in severe major dysmood disorder. Lower NGF and reduced NGF/neurotoxicity ratios were associated with depression severity, suicidal behaviors, recurrence of illness, and adverse childhood experiences.
Experimental animal [103]	Chronic mild stress rat model	↑ IL-1β, TNF-α, IL-6, IL-18; ↓ IL-10, TGF-β; ↑ TNF-α/IL-10 and IL-6/IL-10 ratios	↓ BDNF mRNA in hippocampus and hypothalamus	-	Chronic stress induced a shift toward a pro-inflammatory cytokine profile associated with reduced BDNF expression and decreased hippocampal neurogenesis.
Experimental animal [104]	LPS-induced mouse model of depression with pharmacological manipulation (eFT508; TrkB inhibitor K252a)	Neuroinflammation induced by LPS (elevated inflammatory mediators)	↓ hippocampal BDNF expression and TrkB phosphorylation, ↓ synaptic proteins	-	LPS triggered synaptic protein loss and depressive behaviors via dysregulation of eIF4E-dependent translation and impaired BDNF–TrkB signaling; MNK1/2 inhibition restored BDNF signaling and reduced neuroinflammation; TrkB blockade reverses the effect.
Experimental animal [105]	Chronic stress models in mice with Nlrp1a knockdown	↑ IL-1β, IL-18, IL-6, TNF-α	↓ hippocampal BDNF expression	-	Activation of the NLRP1 inflammasome promoted inflammatory cytokine release and suppressed BDNF signaling via the CXCL1/CXCR2 pathway; Nlrp1a knockdown restored BDNF levels and improved behavior.
Experimental animal [106]	BDNF heterozygous (BDNF^+/−^) mice exposed to LPS	↑ IL-1β, ↑ IL-6, ↑ TNF-α, and microglial activation after LPS	BDNF reduced by ~50% in BDNF^+/−^ mice	-	BDNF deficiency enhanced neuroinflammatory responses (in gender-dependent manner), stronger inflammatory response in BDNF^+/−^ females.
Experimental animal [107]	Comorbid model of chronic pain and depression (DSS-induced colitis + chronic stress)	↑ NF-κB signaling	↓ BDNF mRNA and protein in medial prefrontal cortex; ↓ CREB phosphorylation	-	Microglial loss and NF-κB activation impair BDNF-CREB signaling, linking inflammation with synaptic dysfunction.
Observational clinical study [108]	*n* = 2554 community participants (Dallas Heart Study)	IL-1β ↑, IL-6 ↑, TNF-α ↑, CRP ↑	-	↑ Gal-3	Gal-3 positively predicted depressive symptom severity
Observational clinical study [109]	*n* = 283 patients with type 1 diabetes (30 depressed vs. 253 non-depressed)	-	-	↑ Gal-3 in depressed patients	High Gal-3 is independently associated with depression.
Experimental animal [110]	Gal-3 and Gal-1 knockout mice	-	BDNF unchanged in Gal-3 knockout mice; ↓ BDNF in the prefrontal cortex of Gal-1 knockout	↓ Gal-3 (genetic deletion)	Gal-3 KO mice show depressive-like behavior and compulsive behavior.
***Anxiety***					
Experimental animal [119]	LPS-induced anxiety model (mice); IL-33 knockout mice	↑ IL-33 in basolateral amygdala after LPS; ↑ NF-κB signaling	↓ BDNF in basolateral amygdala		IL-33 suppresses BDNF expression and impairs GABAergic transmission in the amygdala, activating anxiogenic circuits. NF-κB inhibition reverses BDNF suppression and anxiety-like behavior.
Experimental animal [120]	Gal-3^−/−^ mice; LPS-induced neuroinflammation	↓ IL-6, ↓ TNF-α (trend) in hippocampus in Gal-3^−/−^ mice; ↑ IL-6, ↑ TNF-α and TLR4 ↑ in WT + LPS; ↓ IL-6, TLR4 in Gal-3^−/−^ + LPS	↓ hippocampal BDNF in Gal-3^−/−^ mice; LPS BDNF in WT mice + LPS; Gal-3 deletion prevented LPS-induced BDNF decline	-	Gal-3 deficiency induced an anxiogenic behavior under basal conditions, accompanied by reduced hippocampal BDNF levels. In contrast, during LPS-induced neuroinflammation, Gal-3 deletion attenuated anxiety-like behavior, reduced inflammatory cytokine signaling, and preserved BDNF expression, suggesting a context-dependent regulation of anxiety.

SCZ = Schizophrenia; HC = Healthy control; TRS = Treatment-refractory schizophrenia; ECT = electroconvulsive therapy; DSS = Dextran sulfate sodium; eFT508 = Eukaryotic translation initiation factor 4E inhibitor 508; eIF4E = Eukaryotic translation initiation factor 4E; GSK-3β = Glycogen synthase kinase-3 beta; GFAP = Glial fibrillary acidic protein; ↑ = increased; ↓ = decreased.

## 5. Neurodegenerative Diseases

### 5.1. Alzheimer’s Disease

AD is a progressive neurological disorder characterized by memory loss, cognitive decline, and behavioral changes, eventually leading to severe dementia and death. It is associated with abnormal protein deposits in the brain (β-amyloid plaques and tau tangles) that disrupt communication between neurons, and the risk of developing AD increases with age, genetics, and lifestyle factors. AD accounts for about 60–70% of dementia cases worldwide, and with around 50 million people living with dementia, its prevalence is expected to rise significantly as the population ages [121]. Neuroinflammation is increasingly recognized as a key driver of AD pathogenesis, and it may occur years before clinical symptoms appear. Amyloid-β accumulation and tau pathology activate microglia and astrocytes, promoting a reactive state characterized by the release of pro-inflammatory cytokines, reactive oxygen species, and other neurotoxic mediators that contribute to synaptic dysfunction and neuronal loss. Mechanistically, amyloid-β interacts with pattern-recognition receptors on microglia, activating signaling pathways such as NF-κB, MAPK, and inflammasome pathways (e.g., NLRP3), which amplify inflammatory responses and establish a pathological feedback loop that further promotes amyloid production and tau pathology [122,123]. A recent study [124] highlights the importance of neuroinflammatory biomarkers, which indicate glial activation in AD, alongside the role of pro-inflammatory cytokines in further amplifying inflammatory signaling and neuronal injury. In parallel with inflammatory processes, neurotrophic signaling is profoundly disrupted in AD. Reduced BDNF impairs TrkB-dependent pathways that regulate neuronal survival and synaptic plasticity, while BDNF deficiency activates δ-secretase, promoting tau cleavage and generating fragments that further inhibit TrkB signaling and accelerate neuronal degeneration [125]. This vicious cycle contributes to neurofibrillary tangle formation, synaptic loss, and cognitive decline. Furthermore, BDNF also supports synaptic plasticity by facilitating AMPA receptor trafficking, a key mechanism underlying learning and memory, and it promotes myelin integrity by increasing oligodendrocyte progenitor cell density and remyelination capacity [126,127]. Importantly, amyloid-β pathology suppresses BDNF expression by inhibiting CREB-dependent transcription, thereby further amplifying synaptic dysfunction [128]. In contrast to mature BDNF, proBDNF exerts neurodegenerative effects by activating the p75NTR-sortilin receptor complex, which promotes apoptotic signaling and synaptic weakening [129,130]. Similar shifts toward pro-apoptotic signaling are observed in NGF metabolism, where reduced NGF/proNGF ratios impair TrkA signaling and favor proNGF-p75NTR pathways that drive cholinergic degeneration and neuronal loss [131,132,133]. Additional neurotrophins also contribute to disease progression: NT-3 supports hippocampal neuronal survival and synaptic plasticity [134], whereas decreased NT-4/5 levels have been associated with impaired neurogenesis and synaptic dysfunction, both of which are early features of AD pathology [135]. Importantly, these neurotrophic disturbances occur within the background of chronic neuroinflammation, which is characterized by persistent microglial activation and elevated pro-inflammatory cytokines.

Experimental studies (Table 2) provide strong mechanistic evidence linking neuroinflammation with neurotrophic signaling disturbances in Alzheimer’s disease. Specifically, in neuronal cell models of AD, LPS stimulation increased inflammatory mediators, including IL-6 and TNF-α, and elevated expression of cyclooxygenase-2 (COX-2) and nitric oxide synthase (NOS). These inflammatory responses were accompanied by a dose-dependent reduction in BDNF levels and a decrease in neuronal viability, indicating that inflammatory signaling directly suppresses neurotrophic support. Mechanistically, inflammation disrupted intracellular kinase pathways that regulate neuronal survival and synaptic plasticity, including PKA, AKT, and MAPK cascades. At the same time, increased nitric oxide production and oxidative stress further contributed to neuronal dysfunction [136]. In vivo experimental models further support the interaction between inflammatory activation and neurotrophic dysregulation. Cognitive deficits in rats treated with intracerebroventricular administration of amyloid-β25–35 were associated with hippocampal glial activation and elevated levels of cytokines, including IL-1α, IL-1β, and TNF-α. These inflammatory changes were accompanied by reduced NGF expression and decreased transcription of BDNF and GDNF, suggesting impaired neurotrophic support during chronic neuroinflammation [137]. Restoring inflammatory–neurotrophic balance can attenuate neurodegeneration. In an amyloid-beta (Aβ)_1–42_ rat model, TGF-β1 suppressed multiple inflammatory mediators, including TNF-α, IL-1β, IFN-γ, IL-2, IL-17, and IL-22, while preventing declines in BDNF, GDNF, and IGF-1, thereby reducing neuronal injury [138]. Conversely, reduced neurotrophic signaling increases vulnerability to environmental risk factors. In BDNF^^+^/−^ and TrkB^^+^/−^ mice, traumatic brain injury, a high-fat diet, and chronic hypoperfusion enhanced activation of the CCAAT/enhancer-binding protein beta (C/EBPβ-δ) -secretase pathway, increased inflammatory cytokine levels, and accelerated amyloid and tau pathology [139]. Importantly, targeted restoration of neurotrophic signaling can simultaneously modulate neuroinflammation and amyloid pathology. In the 5XFAD mouse model, amyloid-β-specific CD4^+^ T cells engineered to secrete BDNF increased hippocampal TrkB signaling, enhanced synaptic protein expression, reduced IL-1β levels, and decreased amyloid plaque burden [140]. Similarly, in a streptozotocin-induced AD model, apelin-13 suppressed microglial activation and reduced IL-1β and TNF-α, while also restoring BDNF/TrkB signaling and improving cognitive performance [141]. Clinical studies suggest a link between neurotrophic dysregulation and inflammatory activation in AD; however, the findings remain inconsistent due to the limited number of studies and differences in cohorts, disease stage, and methodology. In a cohort including patients with early-onset AD (EOAD), late-onset AD (LOAD), and mild cognitive impairment (MCI), serum BDNF levels were significantly reduced in both EOAD and LOAD patients compared with age-matched controls, while the pro-inflammatory cytokine TNF-α was significantly elevated [142]. Similarly, a comparative clinical study including AD patients, vascular dementia patients, and healthy controls demonstrated significantly lower circulating BDNF levels in AD patients. However, systemic cytokine levels, such as TNF-α and IL-1β, did not differ significantly across groups [143]. These findings suggest that neurotrophic dysregulation may occur even in the absence of marked systemic cytokine elevation. However, other investigations suggest that peripheral BDNF may transiently increase during the early stages of disease, potentially as a compensatory response. In a cohort including AD, mild cognitive impairment, and healthy individuals, AD patients showed significantly elevated plasma BDNF together with increased inflammatory markers, including sTNFR1 and sICAM-1, with sTNFR1 levels strongly associated with disease status and correlated with BDNF concentrations [144]. Interventional evidence further supports the functional coupling of neurotrophic and inflammatory pathways. Specifically, exercise-based interventions in AD patients increased circulating BDNF levels, reduced inflammatory cytokines, and improved cognitive performance [145]. Taken together, these findings indicate that neurotrophic and inflammatory pathways interact dynamically in AD. Disease progression most likely reflects a shifting balance between early compensatory increases in neurotrophic signaling and later neurotrophic depletion associated with chronic neuroinflammation. Therefore, these findings indicate that neurotrophin dysregulation and chronic neuroinflammation form an interconnected pathogenic network in AD, in which inflammatory signaling suppresses BDNF-dependent neuroplasticity, while neurotrophic deficits further amplify neurodegenerative and cognitive processes.

Emerging evidence identifies Gal-3 as an important mediator linking microglial activation with neurodegeneration in AD [52,146,147,148]. Experimental studies [52,146] show that Gal-3 is strongly upregulated in plaque-associated microglia in both human AD brains and transgenic models. Mechanistically, Gal-3 interacts with the microglial receptor TREM2, enhancing downstream inflammatory signaling and promoting amyloid-β pathology. Consistently, genetic deletion of Gal-3 in 5xFAD mice attenuates microglial inflammatory responses, reduces amyloid burden, and improves cognitive performance, supporting a direct role of Gal-3 in AD progression [52]. Proteomic and transcriptomic analyses further identify LGALS3 as one of the most strongly upregulated proteins in disease-associated microglia during amyloid deposition, alongside markers such as APOE, CD68, and CLEC7A, indicating that Gal-3 is a component of a conserved microglial activation program linked to amyloid pathology [147]. Furthermore, experimental studies also demonstrate that immune dysregulation precedes plaque formation. In 5xFAD mice, early microglial alterations involve activation of the JAK/STAT and MAPK pathways, followed by increased cytokine expression, including IL-1β and IL-10, after plaque deposition, suggesting that early innate immune activation contributes to subsequent neurodegeneration [146,148]. Clinical investigations further support the association between Gal-3 and AD. In a cross-sectional study including 57 AD patients and 61 healthy controls, serum Gal-3 levels were significantly elevated in AD, and they increased progressively with disease stage, duration, and age, indicating a potential relationship between Gal-3 and disease severity [149]. Similarly, another case–control study reported significantly higher circulating Gal-3 concentrations in AD patients than in controls, with serum Gal-3 levels correlating with cognitive impairment as measured by the Mini-Mental State Examination (MMSE) [150]. These data indicate that Gal-3 contributes to AD pathogenesis by regulating microglial activation, inflammatory signaling, and amyloid pathology, and may serve as both a biomarker of neuroinflammatory activity and a potential therapeutic target.

Importantly, these findings support a model in which neurotrophin deficiency, chronic neuroinflammation, and galectin-3-mediated microglial activation form a self-reinforcing pathogenic axis that drives synaptic failure, amyloid pathology, and cognitive decline in AD, highlighting the neuroimmune–neurotrophic interface as a promising target for biomarker development and disease-modifying therapies.

### 5.2. Parkinson’s Disease

Parkinson’s disease is the second most common neurodegenerative disorder worldwide, and its prevalence continues to increase with population aging. Moreover, PD is characterized by motor symptoms, including bradykinesia, tremors, and rigidity, as well as a broad spectrum of non-motor manifestations that often precede motor impairment. The immunopathogenesis of PD is progressive degeneration of dopaminergic neurons in the substantia nigra pars compacta, resulting in dopamine depletion within the nigrostriatal pathway [151,152]. PD arises from a complex interaction between genetic susceptibility and environmental exposures. At the molecular level, several interconnected mechanisms contribute to neuronal degeneration, including α-synuclein aggregation, oxidative stress, mitochondrial dysfunction, and neuroimmune activation [153]. Neurotrophic signaling has emerged as an important regulator of dopaminergic neuron survival in PD, with BDNF playing a central role (Table 2). BDNF is essential for the maintenance, plasticity, and survival of nigrostriatal dopaminergic neurons, which are selectively vulnerable in PD [154,155,156,157]. Decreased BDNF levels have been consistently reported in both experimental models and patients with PD, and they correlate with motor symptom severity, cognitive decline, and neuropsychiatric manifestations such as depression [156,158]. The neuroprotective actions of BDNF are primarily mediated through activation of its receptor TrkB, which stimulates intracellular pathways including PI3K/Akt and STAT3 signaling, thereby promoting neuronal survival, autophagy, and resistance to α-synuclein toxicity [156,157,159]. Accordingly, therapeutic strategies aimed at enhancing BDNF/TrkB signaling have demonstrated beneficial effects in several preclinical models. Notably, circulating BDNF levels may vary with disease stage and treatment, and increased BDNF reported in advanced PD likely reflects compensatory responses or levodopa-related effects rather than disease progression itself [160].

Neuroinflammation and neurotrophic signaling are closely interconnected in the pathophysiology of PD. Chronic neuroinflammation is characterized by the activation of microglia and astrocytes and increased production of pro-inflammatory cytokines such as TNF-α, IL-1β, and IL-6. These cytokines activate transcriptional regulators such as NF-κB and YY1, which not only amplify inflammatory responses but also suppress neurotrophic factor expression, including BDNF, in affected brain regions [161,162,163]. The reduction in BDNF is particularly pronounced in the substantia nigra, where dopaminergic neurons are highly vulnerable to inflammatory and oxidative stress [164,165]. Inflammatory mediators further exacerbate neuronal injury through mechanisms involving oxidative stress, excitotoxicity, and activation of innate immune pathways such as the NLRP3 inflammasome, which promotes IL-1β maturation and caspase-1-dependent neurotoxicity [161,163]. Together, these processes establish *a vicious circle* in which inflammation suppresses BDNF signaling, reducing neuronal resilience, while diminished neurotrophic support further increases susceptibility to inflammatory injury. This interaction is additionally modulated by regulatory non-coding RNAs, including miRNAs and the antisense transcript BDNF-AS, which directly influence BDNF transcription and translation [166].

Furthermore, in dopaminergic SH-SY5Y cells exposed to rotenone, activation of the inflammatory transcription factor C/EBPβ markedly suppressed BDNF and netrin-1 expression while promoting δ-secretase activation and pathological processing of α-synuclein and tau. Genetic silencing of C/EBPβ prevented reductions in both BDNF and netrin-1, identifying this pathway as a key transcriptional mechanism linking inflammatory stress to neurotrophic deficiency [167]. Consistent with this concept, systemic LPS administration in mice induces microglial activation in the substantia nigra followed by dopaminergic neuron loss, reduced striatal dopamine levels, impaired motor performance, and decreased BDNF expression. Furthermore, interventions that enhance BDNF signaling, including treadmill exercise or exogenous BDNF administration, protect dopaminergic neurons and restore motor function, whereas pharmacological blockade of TrkB abolishes these protective effects [168]. Similarly, in rotenone-induced PD models, treatment with sericin reduces pro-inflammatory cytokines such as TNF-α and IL-6 while increasing BDNF and TrkB expression in the striatum, resulting in improved motor performance and enhanced antioxidant defenses [169]. In line with these findings, an observational cohort of 104 patients found that early-stage disease was characterized by elevated pro-inflammatory cytokines, such as IL-1β, together with reduced anti-inflammatory mediators, while circulating BDNF levels showed a negative correlation with disease duration, suggesting progressive neurotrophic depletion during disease progression [170]. Alterations in neurotrophic and inflammatory markers are also linked to non-motor manifestations. Cerebrospinal fluid analyses demonstrate that patients with PD and comorbid major depression exhibit reduced levels of both BDNF and IL-6 compared with individuals with depression alone, indicating distinct neuroimmune mechanisms underlying mood disturbances in PD [171]. Interventional evidence further suggests that modulation of neurotrophic signaling can influence inflammatory pathways. In a randomized controlled trial involving PD patients with depression, treatment with *Zishen Pingchan* granules prevented reductions in circulating BDNF, modulated inflammatory cytokines, including IL-1β, IL-6, CRP, and TNF-α, and improved depressive symptoms [172]. Intriguingly, evidence supporting the role of Gal-3 in PD pathogenesis is scarce. A research group led by Boza-Serrano [171,173] found that extracellular α-synuclein induces robust microglial activation, accompanied by increased expression of pro-inflammatory mediators such as IL-1β, IL-12, and iNOS, whereas genetic deletion or pharmacological inhibition of Gal-3 markedly attenuates this response. Consistent with these findings, Gal-3-positive microglia have been observed following uptake of α-synuclein aggregates in vivo, supporting a role for Gal-3 in the transition of microglia toward a disease-associated inflammatory phenotype. Toxin-based PD models further support the pathogenic relevance of this pathway. In the MPTP paradigm, peripheral inflammatory priming with LPS enhances microglial activation, promotes the emergence of Gal-3-expressing disease-associated microglia, and accelerates dopaminergic neuron loss in the substantia nigra. Notably, microglial activation precedes overt neuronal degeneration, suggesting that inflammatory signaling actively contributes to neurodegenerative progression rather than merely reflecting secondary responses [174]. In line with these findings, Yazar and coworkers [175] have reported that elevated circulating Gal-3 levels in patients with PD correlate with disease duration and motor severity, indicating that Gal-3-associated inflammatory activity may reflect ongoing neurodegenerative processes. 

### 5.3. Huntington’s Disease

Huntington’s disease (HD) is a neurodegenerative disease caused by a polyglutamine expansion in the Huntingtin protein, which makes it prone to misfolding and aggregation [176]. The Huntingtin protein serves as a sequestering factor for proteins essential for cellular homeostasis. Neurotrophins also play a crucial role in the pathogenesis and progression of HD, primarily by influencing striatal neuronal survival, synaptic function, and cellular resilience. The depletion of BDNF, resulting from impaired transcription, disrupted axonal transport, and defective secretion, has been consistently implicated as a major driver of striatal degeneration and the associated motor, cognitive, and behavioral dysfunctions characteristic of HD [177,178], as shown in the Table 2. Mutant Huntingtin protein (mHTT) interferes with BDNF gene transcription in cortical neurons, reducing the supply of BDNF to the striatum, which is highly dependent on cortical BDNF for trophic support [178]. Moreover, mHTT impairs BDNF release from astrocytes by disrupting Rab3a-mediated exocytosis, further diminishing local BDNF availability and exacerbating glial dysfunction [179]. This reduction in BDNF leads to selective vulnerability of striatal neurons, as evidenced by region-specific oxidative stress and mitochondrial dysfunction in HD models, which can be mitigated by exogenous BDNF treatment [180]. At the molecular level, low BDNF expression in HD is associated with dysregulation of key signaling pathways, including cAMP, MAPK, and Ras, all of which are simultaneously critical for neuronal survival, synaptic plasticity, and cellular homeostasis [181]. Specifically, the impaired BDNF-TrkB receptor signaling in striatal neurons disrupts corticostriatal synaptic plasticity, contributing to the early synaptic dysfunction and motor symptoms. Notably, this defect is not solely due to reduced BDNF delivery, but it also involves aberrant postsynaptic signaling, which can be partially rescued by targeting p75NTR or PTEN pathways [182]. Furthermore, data from different studies [183,184] revealed that increased BDNF levels, achieved through either recombinant protein delivery or genetic manipulation, have neuroprotective effects in HD models, including improved neuronal survival, reduced striatal atrophy, and amelioration of motor and cognitive deficits [183,184]. In addition to BDNF, other members of the neurotrophin family have also been implicated in HD pathophysiology. Clinical data indicate that patients with HD exhibit reduced circulating, along with increased markers of nitrosative stress, suggesting that a broader range of neurotrophic deficits may accompany disease progression [185]. Experimental studies further demonstrate that NGF supplementation restores hippocampal neurogenesis and improves cognitive performance in HD mouse models [186], whereas NT-3 and NT-4/5 can partially prevent degeneration of striatal projection neurons in toxin-induced HD models, albeit less efficiently than BDNF [187].

Accumulating evidence indicates that neuroinflammatory processes also significantly contribute to HD pathogenesis. Activation of microglia and astrocytes together with increased production of pro-inflammatory cytokines, including IL-6, IL-1β, and TNF-α, has been observed in HD models and patients, often preceding overt neurodegeneration and suggesting that immune dysregulation is an early event in disease progression. In addition, alterations in peripheral immune cells, including hyper-reactive monocytes and macrophages, further support the presence of systemic immune activation in HD [188,189]. Despite the growing recognition that both neurotrophic deficits and neuroinflammatory mechanisms play important roles in HD, relatively few studies have examined their interaction within a unified pathogenic framework. In a quinolinic acid-induced rat model, inhibition of phosphodiesterase-4 with roflumilast attenuated neuroinflammatory activation, reducing TNF-α, IL-6, IFN-γ, and NF-κB levels while restoring BDNF expression in the striatum and cortex through activation of the cAMP/CREB pathway, which was accompanied by reduced oxidative stress and improved motor performance [190]. Complementary observations have been reported by Valadão et al. [191] in genetic HD models. In aged BACHD mice, advanced disease stages were characterized by decreased BDNF and IL-6, together with increased MCP-1 and NGF levels in the striatum, indicating an imbalance between inflammatory mediators and neurotrophic signaling that may reflect compensatory responses during disease progression.

Despite increasing recognition of neuroinflammatory mechanisms in HD progression, studies specifically addressing the role of Gal-3 in this disease remain relatively limited. Nevertheless, available experimental evidence indicates that Gal-3 is an important regulator of microglia-mediated inflammatory responses in HD. In HD models, Gal-3 expression is markedly increased in activated microglia, where it acts as a sensor of lysosomal damage and promotes inflammatory signaling through NF-κB-dependent pathways and inflammasome activation. Genetic suppression of Gal-3 in the R6/2 HD mouse model attenuates microglial activation, reduces pro-inflammatory cytokine production, and improves motor performance and survival, supporting a direct contribution of Gal-3-dependent microglial signaling to disease progression [192]. More recent work has begun to explore the therapeutic implications of targeting this pathway. Using a high-content screening strategy based on Gal-3 puncta formation as a readout of lysosomal damage sensing, a brain-penetrant small-molecule inhibitor, berbamine hydrochloride, was identified as a potent modulator of intracellular Gal-3 activity. In the R6/2 mouse model of HD, treatment with berbamine hydrochloride reduced microglial activation, decreased mutant Huntingtin aggregation, restored neuronal signaling markers such as DARPP-32, and improved motor performance. Transcriptomic analyses further identified Gal-3 as a central hub within HD-associated transcriptional networks that were partially normalized following treatment [193].

Together, these findings position Gal-3 as a key molecular node linking microglial inflammation with neuronal dysfunction in HD. However, despite the established contribution of both neurotrophic deficits and neuroinflammatory signaling to HD pathology, studies examining their interaction, particularly in the context of Gal-3-mediated immune activation, remain scarce.

**Table 2:** Experimental and clinical studies linking neurotrophins, inflammation, and Gal-3 in neurodegenerative disorders.

Study Design	Sample/Model	Key Inflammatory Markers	Neurotrophin Levels	Gal-3 Levels	Main Findings
***Alzheimer’s Disease***					
Experimental in vitro [136]	SH-SY5Y neuronal cells; LPS-induced neuroinflammation (2D and 3D spheroid cultures)	↑ IL-6, ↑ TNF-α, ↑ COX-2, ↑ NOS, ↑ oxidative stress	↓ BDNF	-	LPS-induced neuroinflammation suppresses BDNF signaling and neuronal viability by disrupting intracellular survival pathways.
Experimental animal model [137]	Rat AD model; intracerebroventricular Aβ25-35 administration	↑ IL-1α, ↑ IL-1β, ↑ TNF-α; ↑ GFAP; ↑ Iba1	↓ hippocampal NGF; ↓ hippocampal BDNF/GDNF transcription; transient ↑ BDNF/GDNF proteins	-	Aβ-induced neuroinflammation disrupts the cytokine–neurotrophin balance and contributes to neuronal dysfunction.
Experimental animal model [138]	Aβ_1–42_ rat model (intrahippocampal injection)	↑ TNF-α, ↑ IL-1β, ↑ IFN-γ, ↑ IL-2, ↑ IL-17, ↑ IL-22, ↑ iNOS	↓ hippocampal BDNF (restored by TGF-β1)	-	TGF-β1 suppressed neuroinflammation and restored neurotrophic signaling, attenuating Aβ-induced neurodegeneration.
Experimental animal model [139]	BDNF^+/−^ and TrkB^+/−^ mice exposed to environmental stressors	↑ IL-1β, IL-6, TNF-α; activation of C/EBPβ-δ-secretase pathway	↓ BDNF/TrkB signaling (genetic deletion)	-	Neurotrophic deficiency increased susceptibility to inflammation-driven Aβ and tau pathology.
Experimental animal model [140]	5XFAD AD mice; intracerebroventricular injection of Aβ-specific CD4^+^ T cells expressing BDNF	↓ IL-1β; ↑ SIRP-β1 (phagocytic marker)	↑ BDNF; ↑ TrkB activation	-	BDNF-producing T cells targeted amyloid plaques, increased neurotrophic signaling, reduced amyloid pathology, and promoted synaptic repair.
Experimental animal model [141]	STZ-induced sporadic AD rat model; hippocampal analysis after apelin-13 treatment	↑ IL-1β, ↑ TNF-α, ↑ IBA1 (microglia), ↑ GFAP (astrocytes)	↓ hippocampal BDNF and p-TrkB (restored by apelin-13)	-	Apelin-13 suppressed glial activation and pro-inflammatory cytokines, restored BDNF/TrkB signaling, and improved synaptic markers and cognition; these effects were blocked by a TrkB antagonist.
Observational clinical study [142]	EOAD (*n* = 22), LOAD (*n* = 50), MCI (*n* = 30) patients	TNF-α ↑	↓ BDNF	-	Neurotrophic depletion accompanied by inflammatory activation.
Observational clinical study [143]	AD (*n* = 60), vascular dementia (*n* = 60), controls (*n* = 33)	TNF-α, IL-1β unchanged	↓ BDNF	-	Reduced BDNF in AD independent of systemic cytokine changes.
Observational clinical study [144]	AD (*n* = 50), MCI (*n* = 37), controls (*n* = 56)	sTNFR1 ↑, sICAM-1 ↑	↑ BDNF	-	Elevated BDNF is associated with inflammatory activation.
Interventional clinical study [145]	AD patients undergoing exercise intervention (*n* = 15)	↓ IL-2	↑ BDNF	-	Exercise increased BDNF and reduced inflammation with cognitive improvement
Experimental animal [52]	5xFAD mice and primary microglia	5xFAD mice and primary microglia	-	↑ microglial Gal-3	Gal-3 amplifies microglial activation and amyloid pathology; Gal-3 deletion reduces inflammation, amyloid burden, and cognitive deficits.
Experimental animal [147]	APPPS1 and APP-KI AD mouse models	↑ microglial immune activation pathways	-	↑ brain microglial Gal-3	Gal-3 was identified as an early microglial amyloid-response protein associated with disease-associated microglia during amyloid deposition.
Experimental animal [148]	5xFAD mice	↑ IL-1β, ↑ IL-10; activation of JAK/STAT and MAPK pathways	-	↑ brain microglial Gal-3	Early innate immune activation occurs in microglia prior to plaque deposition, indicating neuroinflammatory changes in AD.
Observational clinical study [149]	57 AD patients, 61 controls	CRP unchanged	-	↑ serum Gal-3	Gal-3 levels were significantly higher in AD patients and increased with disease stage, age, and duration.
Observational clinical study [150]	41 AD, 32 MCI, 46 controls	-	-	↑ serum Gal-3	Higher Gal-3 in AD patients correlated with MMSE scores, suggesting an association with cognitive decline.
***Parkinson’s disease***					
Experimental animal [167]	Rotenone-treated dopaminergic SH-SY5Y cells and PD mouse models	↑ inflammatory signaling via C/EBPβ and oxidative stress	↓ BDNF in the substantia nigra	-	C/EBPβ activation represses BDNF expression, linking inflammation to neurotrophic deficiency in PD.
Experimental animal [168]	LPS-induced neuroinflammation mouse model	↑ IL-1β, ↑ TNF-α, ↑ IL-6, microglial activation	↓ BDNF in substantia nigra; exercise restores BDNF-TrkB signaling	-	Exercise protects dopaminergic neurons from LPS-induced degeneration via activation of BDNF-TrkB signaling.
Experimental animal [169]	Rotenone-induced PD rat model; sericin treatment.	↑ TNF-α, ↑ IL-6	↓ BDNF and TrkB in the striatum area; sericin treatment restores BDNF/TrkB	-	Sericin reduces inflammation and restores BDNF/TrkB signaling, improving motor deficits in rotenone-induced PD.
Observational clinical study [170]	104 PD patients	↑ IL-1β; ↓ IL-5, ↓ IL-10, ↓ IL-17 in early PD	BDNF negatively correlated with disease duration	-	Early PD shows a pro-inflammatory profile, while BDNF levels decline with disease progression.
Observational clinical study [171]	PD with MDD (*n* = 11), PD without depression (*n* = 14), MDD (*n* = 12); CSF biomarker accesment	↓ IL-6 in PD with depression	↓ BDNF in PD with depression after treatment	-	PD with depression shows altered CSF BDNF and IL-6 regulation compared with depression alone.
Randomized double-masked placebo-controlled trial [172]	80 PD patients with depression; *Zishen pingchan* treatment	IL-1β, IL-6, TNF-α, IL-2, IFN-γ, IL-17	*Zishen pingchan* treatment prevented decline in BDNF	-	Improvement of depressive symptoms in PD is associated with preservation of BDNF levels and modulation of inflammatory cytokines.
Experimental animal [173]	α-synuclein–stimulated microglia; mouse PD models	↑ IL-1β, ↑ IL-12, ↑ iNOS	-	↑ brain microglial Gal-3	Gal-3 promotes α-synuclein-induced microglial inflammation
Experimental animal [174]	MPTP mouse model with LPS priming	↑ IL-1β, ↑ TNF-α, ↑ IL-6	-	↑ brain microglial Gal-3	Gal-3-associated microglia accelerate dopaminergic neurodegeneration.
Observational clinical study [175]	48 PD patients, 63 controls	-	-	↑ serum Gal-3	Elevated Gal-3 correlates with PD severity and disease duration.
***Huntington disease***					
Experimental animal [190]	Quinolinic acid-induced rat model of HD; roflumilast treatment.	↑ TNF-α, IL-6, IFN-γ, NF-κB (reduced after treatment)	↓ BDNF in the striatum and cortex of the rat brain (restored after treatment via cAMP/CREB pathway)	-	Roflumilast treatment attenuated neuroinflammation, restored BDNF signaling, and improved motor performance.
Experimental animal [191]	24-month-old BACHD transgenic mice	IL-6, ↑ MCP-1 in striatum	↓ BDNF, ↑ NGF in striatum; ↑ BDNF in prefrontal cortex	-	Advanced HD shows imbalance between inflammatory mediators and neurotrophic factors, suggesting region-specific compensatory responses.
Experimental animal [192]	R6/2 HD mouse model	↑ IL-1β, ↑ IL-6, ↑ TNF-α; ↑ microglial activation markers (Iba1, Cd68)	-	↑ brain microglia Gal-3	Gal-3 promotes microglia-driven neuroinflammation and HD progression; Gal-3 suppression reduces cytokine production and improves motor outcomes.
Experimental animal [193]	R6/2 HD mice treated with berbamine hydrochloride	↓ TNF-α; ↓ microglial activation (Cd68); ↓ inflammatory transcriptional networks	-	↓ Gal-3 expression/activity (Gal-3 inhibitor used)	BBB-penetrant Gal-3 inhibitor attenuates neuroinflammation, reduces mHTT aggregation, and improves motor performance.

↑ = increased; ↓ = decreased; APPPS1 = Amyloid precursor protein/presenilin 1 transgenic model; APP-KI = Amyloid precursor protein knock-in; CSF = Cerebrospinal fluid; Iba1 = Ionized calcium-binding adapter molecule 1.

## 6. Other Neurological Disorders

### 6.1. Multiple Sclerosis

MS presents a chronic autoimmune disease of the CNS characterized by inflammation, demyelination, gliosis, and progressive neuronal loss. The disease primarily targets myelinated axons, leading to relapsing neurological deficits and long-term neurodegeneration, and affects approximately 2.3 million individuals worldwide, with higher prevalence in women and populations of Northern European ancestry [194].

Neurotrophins, particularly BDNF and NGF, play important roles in MS immunopathogenesis and tissue repair [195,196,197,198]. BDNF, the most extensively studied neurotrophin in this disease, is produced by neurons, glial cells, and immune cells and supports recruitment, maturation, and survival of oligodendrocyte precursor cells required for myelin repair [195,197]. Published data reported that BDNF expression within MS lesions protects axons and mitigates disease severity, whereas BDNF deficiency accelerates axonal loss and disability progression [199]. NGF also contributes to myelin repair by promoting axonal regeneration, oligodendrocyte differentiation, and migration of oligodendrocyte precursor cells to demyelinated regions. In addition, NGF regulates myelin proteins and induces BDNF expression, highlighting coordinated neurotrophin signaling in CNS repair [198]. However, neurotrophin signaling in MS is context-dependent. While BDNF and NGF generally exert neuroprotective effects, activation of astrocytic TrkB receptors within MS lesions can induce nitric oxide production, which may contribute to neurodegeneration, whereas mice lacking astrocytic TrkB are protected from experimental autoimmune encephalomyelitis (EAE)-induced injury [200]. Autoreactive T cells may also release BDNF, potentially supporting tissue repair while simultaneously enhancing survival of pathogenic lymphocytes [201]. BDNF, through TrkB signaling, promotes oligodendrocyte progenitor maturation and increases myelin sheath thickness, thereby facilitating remyelination [201,202,203,204]. Consistent with this concept, some disease-modifying therapies, such as glatiramer acetate, may partly exert neuroprotective effects by promoting neurotrophin release from immune cells that cross the blood–brain barrier [201]. Neuroinflammatory processes also play a central role in MS pathogenesis and progression. Infiltration of autoreactive immune cells into the CNS, particularly T and B lymphocytes, together with the activation of microglia and macrophages, promotes the production of pro-inflammatory mediators, including IL-1β, IL-17, IFN-γ, and TNF-α, which contribute to demyelination and axonal injury [205]. Inflammatory cells also generate reactive oxygen and nitrogen species that amplify oxidative stress, mitochondrial dysfunction, and neuronal damage within MS lesions [206]. These mechanisms indicate that chronic neuroinflammation not only drives immune-mediated demyelination but also disrupts neurotrophic support, thereby contributing to progressive neurodegeneration. In the cuprizone-induced demyelination model, increased brain levels of the pro-inflammatory cytokine TNF-α are accompanied by reduced BDNF expression, suggesting that inflammatory activation is associated with impaired neurotrophic support during demyelination. Interestingly, serum levels of both TNF-α and BDNF increase during the early phase of cuprizone exposure, indicating differential central versus peripheral regulation of neurotrophin–inflammatory crosstalk during disease progression and recovery [207]. Similarly, in EAE, BDNF produced by infiltrating immune cells within inflammatory lesions contributes to axonal protection during autoimmune demyelination, demonstrating that immune-derived neurotrophins can directly modulate neuronal survival under neuroinflammatory conditions [199]. Immunomodulatory therapy with glatiramer acetate further enhances expression of neurotrophins such as BDNF, NT-3, and NT-4 in the CNS while promoting anti-inflammatory cytokine production by infiltrating T cells, highlighting that immune modulation can stimulate endogenous neuroprotective and repair pathways [208].

Moreover, clinical studies (Table 3) provide additional support for this neurotrophin–inflammation interaction. Immunohistochemical analyses of MS brain tissue demonstrate that BDNF is expressed not only by neurons but also by infiltrating immune cells, including macrophages/microglia and T lymphocytes, as well as reactive astrocytes within demyelinating plaques. The proportion of BDNF-positive immune cells is higher in actively demyelinating lesions than in inactive plaques, indicating that inflammatory activity is associated with increased neurotrophin production within the lesion microenvironment [209]. In addition, peripheral immune cells also contribute to neurotrophin dynamics. In a clinical study involving 74 MS patients, serum BDNF levels were significantly elevated compared with controls, and they were further increased in individuals receiving interferon-β therapy. Interferon-β also enhanced BDNF production by T cells and increased TrkB receptor expression in peripheral blood mononuclear cells, suggesting that immunomodulatory therapy may promote endogenous neuroprotective pathways [210]. The balance between neurotrophic and inflammatory signaling also appears to influence clinical manifestations of MS. In relapsing–remitting disease, reduced BDNF production by peripheral blood mononuclear cells is associated with impaired cognitive performance, whereas elevated IL-6 levels correlate with lower MMSE scores, indicating that diminished neurotrophic support, combined with pro-inflammatory signaling, may contribute to cognitive impairment [211]. In progressive MS, circulating IL-6 levels were increased while BDNF levels remained unchanged, resulting in a markedly reduced BDNF/IL-6 ratio. Notably, a higher BDNF/IL-6 ratio correlates with improved walking speed, lower fatigue, and greater aerobic fitness, suggesting that this balance may represent a potential functional biomarker of disease status [212]. Consistent with this concept, a pilot clinical trial of testosterone therapy demonstrated immunomodulatory effects characterized by reduced CD4^+^ T cells, increased NK cells, decreased IL-2 production, and increased TGF-β1 levels. During treatment, peripheral immune cells produced substantially higher levels of neurotrophic factors, including BDNF and PDGF-BB, indicating that immune cell-mediated neurotrophin production may contribute to neuroprotection during inflammatory responses [213]. Importantly, evidence regarding the role of Gal-3 in MS is still limited, but it suggests that this lectin regulates both inflammatory and reparative processes. Thus, in the cuprizone model, demyelination increases Gal-3 expression together with CD45^+^/Iba1^+^ microglial accumulation in the corpus callosum, while the adjacent subventricular zone shows reduced numbers of Gal-3^+^ and CD45^+^ cells. Gal-3 deficiency disrupts progenitor responses and impairs the coordinated reaction to demyelination, indicating that Gal-3 contributes to remyelination by modulating inflammatory signaling in lesioned white matter and neurogenic niches [214]. Consistently, Gal-3-deficient mice exposed to cuprizone exhibit impaired remyelination, abnormal myelin architecture, reduced MMP-3 induction, and the persistence of CD45^+^, TNF-α^+^, and TREM-2b^+^ cells, indicating that Gal-3 is required for appropriate microglial activation and efficient myelin repair [215]. In contrast, in EAE, Gal-3 appears to promote disease, as its deficiency reduces clinical severity, decreases CNS leukocyte infiltration and IL-17/IFN-γ production, and increases IL-10, IL-5, IL-13, and Foxp3^+^ regulatory T cells [216]. Clinical observations further support the relevance of Gal-3 in MS pathology. Proteomic screening identified autoantibodies against Gal-3 in the sera of 10 of 11 patients with secondary progressive MS, whereas such antibodies were not detected in other neurological diseases or healthy controls. Functional analyses demonstrated that these antibodies induce ICAM-1 upregulation and NF-κB p65 phosphorylation in brain microvascular endothelial cells, indicating that anti-Gal-3 immune responses may contribute to blood–brain barrier disruption and persistent neuroinflammation in progressive MS [217].

### 6.2. Stroke

Stroke leads to a cascade of inflammatory reactions and is a leading cause of mortality and disability. The American Heart Association defines stroke as an acute neurological impairment caused by vascular issues, encompassing ischemia as well as intracerebral and subarachnoid hemorrhages [218]. Beyond the first few hours following a stroke, conventional therapy is directed toward medical thrombolysis and mechanical thrombectomy, which have impressive results; however, no treatment is available once the ischemic injury has been established, and therefore, regenerative approaches are being intensively examined [219].

Neurotrophins have important roles in stroke pathophysiology and recovery by activating signaling pathways such as PI3K/AKT and MAPK, which support neuronal survival, inhibit apoptosis, and promote axonal regeneration and angiogenesis. In the acute phase of ischemic injury, they exert cytoprotective effects by reducing neuronal apoptosis and modulating inflammatory responses [220,221,222,223,224]. BDNF/TrkB signaling is particularly critical for neuronal survival under hypoxic conditions and the maintenance of neuronal viability within the ischemic penumbra [221,224]. After a stroke (Table 3), an imbalance occurs in which protective BDNF-TrkB signaling decreases while pro-apoptotic proBDNF/p75NTR signaling increases, contributing to neuronal loss and adverse outcomes [225]. Ramos-Cejudo and coworkers [226] show that exogenous BDNF enhances oligodendrocyte differentiation, remyelination, and white matter repair, hence improving functional recovery. Furthermore, stroke patients typically exhibit lower circulating BDNF levels, although aerobic exercise can acutely increase BDNF concentrations and may support neuroplasticity and recovery [227,228]. Importantly, these neurotrophic mechanisms operate within a highly inflammatory microenvironment generated by ischemic injury. Neuroinflammation is rapidly initiated after a stroke through the activation of resident glial cells and the recruitment of peripheral immune cells, leading to the release of pro-inflammatory cytokines, chemokines, and reactive oxygen species that contribute to blood–brain barrier disruption and secondary neuronal damage [229]. The inflammatory cascade further evolves through interactions among innate and adaptive immune responses and thrombo-inflammatory processes, which amplify tissue injury and represent potential therapeutic targets for modulating post-stroke recovery [230]. Recent clinical studies further support the interaction between neurotrophic signaling and inflammatory pathways in acute ischemic stroke. In a prospective study of 42 AIS patients, both MMP-9 and BDNF levels were elevated during the acute phase compared with controls, reflecting concurrent activation of inflammatory injury and neuroplastic responses. Importantly, persistent elevation of MMP-9, together with declining BDNF levels over time, was associated with poorer 3-month functional outcomes, whereas decreasing MMP-9 and increasing BDNF predicted improved recovery [231]. Additional evidence highlights the prognostic relevance of inflammatory mediators and blood–brain barrier injury markers. In a cohort of 138 patients with first-ever ischemic stroke, higher levels of IL-6, TNF-α, CRP, leukocytes, and S100B were significantly associated with more severe neurological deficits and less favorable functional outcomes. In contrast, BDNF did not show a significant association with stroke severity or short-term prognosis. A positive correlation between BDNF and VEGF was also observed, suggesting a potential link between neurotrophic signaling and post-ischemic angiogenic responses [232]. Moreover, BDNF regulates post-ischemic inflammation at cellular, cytokine, and transcriptional levels. In a rat MCAO model, exogenous BDNF improved sensorimotor and vestibulomotor function while increasing the number of activated and phagocytic microglia in ischemic brain tissue. Additionally, BDNF upregulated the anti-inflammatory cytokine IL-10 and suppressed the pro-inflammatory cytokine TNF-α, while enhancing NF-κB DNA-binding activity [233].

In parallel with neurotrophins, increasing evidence highlights Gal-3 as a key mediator linking inflammation, tissue injury, and neurological outcome after stroke. Clinical studies consistently demonstrate that circulating Gal-3 levels are elevated in patients with ischemic stroke, and they correlate with both the stroke severity and poor functional outcomes. In patients with large-artery atherosclerotic stroke, serum Gal-3 levels independently predicted unfavorable functional outcome at 90 days [234]. Similar findings were observed in larger cohorts, in which higher Gal-3 concentrations correlated with NIHSS scores and systemic inflammatory markers, such as CRP [235]. Moreover, elevated Gal-3 levels have been associated with infarct volume, neurological severity, and poor long-term recovery in patients with first-ever ischemic stroke [236]. In experimental models, Gal-3 expression increases rapidly after brain injury, predominantly in microglia, where it interacts with TLR-4 signaling to promote M1 polarization and the production of pro-inflammatory cytokines, including IL-1β, TNF-α, and IL-6 [237]. Downregulation of Gal-3 reduces neuronal apoptosis, oxidative stress, blood–brain barrier disruption, and cerebral edema, ultimately improving neurological recovery [236]. Consistently, pharmacological inhibition of Gal-3 reduces infarct size, neuronal apoptosis, and microglial activation while suppressing NLRP3 inflammasome signaling through the TLR4/NF-κB pathway [238]. Taken together, these findings indicate that stroke outcome is determined by a complex interplay between neurotrophic repair mechanisms and inflammatory injury pathways. Understanding the balance between neurotrophic signaling, particularly BDNF-mediated pathways, and inflammatory mediators such as Gal-3 may provide important insights for the development of novel biomarkers and targeted therapeutic strategies aimed at improving post-stroke recovery.

**Table 3:** Experimental and clinical studies linking neurotrophins, inflammation, and Gal-3 in multiple sclerosis and stroke.

Study Design	Sample/Model	Key Inflammatory Markers	Neurotrophin Levels	Gal-3 Levels	Main Findings
***Multiple sclerosis***					
Experimental animal [207]	Cuprizone-induced demyelination model (C57BL/6 mice)	↑ TNF-α in the brain	↓ BDNF in brain; ↑ BDNF in serum (early phase)	-	Neuroinflammation is associated with reduced central BDNF during demyelination, indicating crosstalk between inflammatory signaling and neurotrophic support.
Experimental animal [199]	MOG-induced EAE mouse model	↑ T cells, macrophages	↑ BDNF (immune cells of brain lesions)	-	Immune-derived BDNF protects axons and reduces neurodegeneration during autoimmune demyelination.
Experimental animal [208]	EAE mice treated with glatiramer acetate	↓ IFN-γ, ↑ IL-10, TGF-β	↑ BDNF, ↑ NT-3, ↑ NT-4 (brain)		Immunomodulation enhances neurotrophin expression and promotes neuroprotection and repair.
Clinical observational study [209]	Post-mortem MS brain tissue (9 MS patients; 5 controls)	↑ CD3^+^ T cells, CD68^+^ microglia	↑ BDNF (immune cells and astrocytes in brain lesions)	-	A higher proportion of BDNF^+^ immune cells in active demyelinating lesions suggests a neuroprotective immune response.
Clinical observational study + in vitro [210]	74 MS patients, 32 healthy controls; serum + PBMC assays	Not directly measured	↑ serum BDNF ↑; further ↑ with IFN-β; TrkB expression ↑ in PBMCs	-	IFN-β enhances BDNF production by immune cells, indicating therapy-induced neuroprotective signaling.
Clinical observational study + in vitro [211]	30 relapsing–remitting MS patients vs. matched controls	↑ IL-10; IL-6 n.s., TNF-α n.s.	↓ BDNF (PBMC)	-	↓ BDNF and ↑ IL-6 are associated with worse cognitive performance.
Clinical observational study [212]	14 progressive MS patients + 7 matched controls; exercise test	↑ IL-6; ↑ IL-6 after exercise	BDNF n.s.; ↓ BDNF/IL-6 ratio	-	↑ BDNF/IL-6 ratio associated with better walking speed, lesser fatigue, and greater aerobic fitness.
Clinical trial [213]	10 male relapsing–remitting MS patients treated with testosterone for 12 months; PBMC assays	↓ IL-2, CD4^+^ T cells; ↑ NK cells, TGF-β1	↑ BDNF	-	Testosterone induces an anti-inflammatory immune shift and increases neurotrophic factor production by immune cells.
Experimental animal [214]	Cuprizone-treated WT and Gal-3 knockout mice	↑ CD45^+^/Iba1^+^ microglia in corpus callosum; ↓ Gal-3^+^ and CD45^+^ cells in t subventricular zone	-	↑ Gal-3 in the corpus callosum	Gal-3 regulates region-specific inflammatory responses and contributes to remyelination after cuprizone injury.
Experimental animal [215]	Cuprizone-treated WT and Gal-3^−/−^ mice	Persistence of CD45^+^, TNF-α^+^, and TREM-2b^+^ cells in Gal-3-deficient mice; impaired microglial tuning	-	↓ Gal-3 (genetic deletion)	Gal-3 supports myelin repair in toxin-induced demyelination.
Experimental animal [216]	MOG35–55-induced EAE in WT and Gal-3^−/−^ mice	↓ CNS leukocyte infiltration, ↓ IL-17, ↓ IFN-γ; ↑ IL-10, ↑ IL-5, ↑ IL-13; ↑ Foxp3^+^ Treg cells in Gal-3^−/−^ mice	-	↓ Gal-3 (genetic deletion)	In autoimmune demyelination, Gal-3 promotes pathogenic inflammation and worsens disease.
Clinical observational study + in vitro [217]	27 MS patients (11 secondary progressive MS, 16 relapsing–remitting MS), disease controls, and healthy subjects	↑ ICAM-1, phospho-NF-κB p65 (endothelial cells exposed to secondary progressive MS sera)	-	Anti-Gal-3 antibodies were detected in 10/11 SPMS patients	Autoantibodies targeting Gal-3 promote endothelial activation and may contribute to BBB disruption in secondary progressive MS.
***Stroke***					
Clinical observational study [231]	Acute ishemic stroke patients (*n* = 42) vs. healthy controls (*n* = 40); biomarkers measured Day 1, Day 2, Day 5	↑ MMP-9	↑ BDNF; dynamic change over time	-	↑ Baseline MMP-9 and sustained increase predicted worse 3-month outcome, while ↑ BDNF and ↓ MMP-9 were associated with better functional recovery.
Clinical observational study [232]	First-ever ischemic stroke patients (*n* = 138)	↑ IL-6, TNF-α, CRP, WBC, S100B	BDNF n.s.	-	Elevated inflammatory markers correlated with higher NIHSS and worse 30-day mRS outcomes; BDNF correlated with VEGF, suggesting a link between neurotrophic and angiogenic responses.
Experimental animal [233]	Experimental stroke model; rat MCAO; exogenous BDNF	↑ IL-10, NF-κB, ↓ TNF-α (after exogenous BDNF)	↑ BDNF (exogenous administration)	-	BDNF increased the number of activated and phagocytic microglia, shifted the cytokine profile toward anti-inflammatory signaling, reduced neuronal apoptosis, and improved neurological function.
Clinical observational study [234]	130 patients with large-artery atherosclerotic stroke + 130 controls	↑ CRP	-	↑ Gal-3	↑ Gal-3 independently predicted an unfavorable 90-day outcome.
Clinical observational study [235]	288 patients with first-ever acute ischemic stroke	↑ CRP	-	↑ Gal-3	Gal-3 correlated with NIHSS severity and predicted poor functional outcome.
Clinical observational study [236]	233 acute ischemic stroke patients + 252 controls	-	-	↑ Gal-3	Gal-3 is associated with infarct severity and long-term functional outcome; Gal-1 is not predictive.
Experimental animal [237]	Rat intracerebral hemorrhage model	↑ IL-1β, TNF-α, IL-6; ↑ M1 microglia; ↑ ROS	-	↑ brain microglia Gal-3	Gal-3 promotes microglia-mediated neuroinflammation and worsens brain injury after ICH, while its inhibition reduces inflammation and improves neurological outcomes.
Experimental animal + in vitro [238]	Ischemia/reperfusion injury in mice; Gal-3 inhibitor	↑ NLRP3, cleaved-caspase-1, IL-1β (after injury); ↓ TLR4/NF-κB signaling (Gal-3 inhibitor)	-	↑ brain microglia Gal-3	Pharmacological Gal-3 inhibition reduced infarct size, neuronal apoptosis, and neuroinflammation by suppressing the Gal-3/TLR4/NF-κB/NLRP3 pathway.

↑ = increased; ↓ = decreased.

## 7. Mechanistic Insights, Therapeutic Implications, and Limitations

A growing body of evidence indicates that neurotrophins and Gal-3 should not be considered as independent mediators, but rather as interlinked components of a broader neuroimmune regulatory network. The reported data support a theory in which inflammatory signaling, neurotrophic support, and microglial activation converge to shape neuronal function and disease progression. Pro-inflammatory cytokines, such as TNF-α, IL-1β, and IL-6, suppress neurotrophin signaling through NF-κB, MAPK, and inflammasome-dependent pathways, leading to reduced neurotrophin expression, impaired synaptic plasticity, and increased neuronal vulnerability. Additionally, Gal-3 acts as an upstream amplifier of neuroinflammation by promoting microglial activation through receptors TREM2 and TLR4, as well as downstream signaling pathways including PI3K/Akt, NF-κB, and NLRP3 inflammasome activation. These processes enhance cytokine production and sustain a pro-inflammatory milieu within the CNS. Importantly, these mechanisms form a reciprocal cycle in which Gal-3-driven inflammatory signaling suppresses neurotrophin-mediated neuronal support, until reduced neurotrophic activity further increases susceptibility to inflammatory damage. Hence, Gal-3 primarily functions as a regulator of immune activation, whereas neurotrophins act as key determinants of neuronal resilience and repair. The balance between these systems appears to be highly dependent and affected by disease stage, cellular composition, and receptor dynamics.

Current neurotrophin-based drug development is shifting toward synthetic microforms and engineered ligands. These advanced platforms aim to optimize therapeutic efficacy, specifically targeting neuronal regeneration and plasticity more effectively than endogenous neurotrophins [239,240,241,242,243,244]. Moreover, inhibition of Gal-3 has demonstrated anti-inflammatory and neuroprotective effects in preclinical models by attenuating microglial activation, inflammasome signaling, and oxidative stress [245,246,247,248,249]. Collectively, these findings support the concept that simultaneous modulation of neurotrophic and inflammatory pathways may offer greater therapeutic benefit than single-target approaches. However, clinical translation of these strategies remains significantly limited by delivery-related challenges. Furthermore, neurotrophins exhibit poor BBB permeability due to their large molecular size, hydrophilicity, and unfavorable pharmacokinetic profiles, including rapid systemic clearance and restricted tissue diffusion [250,251,252,253]. Similarly, most Gal-3 inhibitors, particularly carbohydrate-based compounds, display limited BBB penetration and may be further constrained by active efflux mechanisms and insufficient intracellular target engagement [193,254,255]. To address these limitations, advanced delivery strategies such as receptor-mediated transcytosis, nanoparticle-based systems, intranasal administration, and transient BBB modulation are actively being explored [253,256,257,258]. Recent development of BBB-penetrant Gal-3 inhibitors with improved pharmacokinetic properties further highlights the potential of rational drug design in overcoming these barriers [193,254]. Nevertheless, efficient CNS delivery and sustained target engagement remain critical challenges. In addition, the effects of both neurotrophins and Gal-3 raise concerns regarding potential off-target or disease stage responses. Finally, the limited availability of well-controlled clinical studies underscores the need for rigorous translational validation, including optimization of dosing, safety, and long-term efficacy.

## 8. Conclusions and Future Directions

This review integrates current evidence indicating that neurotrophins and Gal-3 represent functionally interlinked components of the neuroimmune regulatory network that critically shape neuronal function in both neurodegenerative and psychiatric disorders. The available data support a model of microglial activation through Gal-3 in which inflammatory signaling suppresses neurotrophin-mediated neuronal support, thereby contributing to impaired synaptic plasticity, reduced neurogenesis, and increased vulnerability to neurodegeneration.

Despite these advances, several important limitations remain. A substantial proportion of the available evidence is derived from peripheral biomarkers, while mechanisms within the CNS are insufficiently characterized. In addition, direct mechanistic studies linking neurotrophins and Gal-3 are limited, and current interpretations rely largely on indirect associations. Therefore, future research should focus on region and specific cell type, especially in microglia and astrocytes, analyses within the CNS, identification of shared intracellular signaling pathways connecting neurotrophins and Gal-3, and validation of this axis in longitudinal and disease stage-specific models. Combined biomarker strategies that include Gal-3 and neurotrophins in cerebrospinal fluid or peripheral blood may also enhance disease detection, prognosis, and monitoring of treatment responses in neurodegenerative and neuropsychiatric disorders. Moreover, combination therapies, such as combining Gal-3 inhibitors with neurotrophin mimetics or agents that boost natural neurotrophic signaling, should be investigated, particularly in age-related neurological diseases where inflammation and neurotrophic deficits occur together. Finally, translational studies and clinical trials are needed to validate these strategies, optimize delivery systems, and develop context-specific interventions based on disease stage and cellular targets.

**Figure 2:**
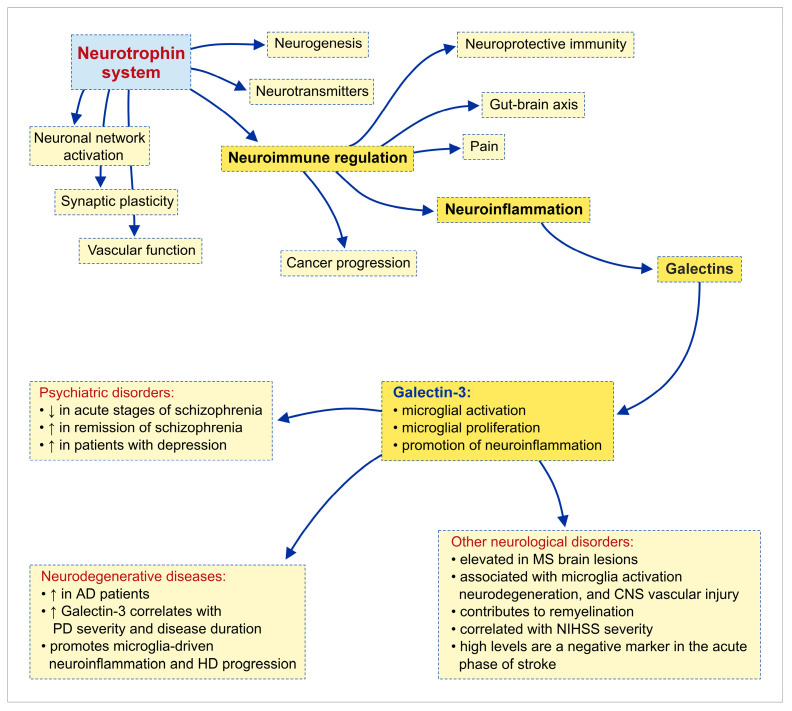
The effects of neurotrophins: Gal-3 axis in neuroinflammation-mediated diseases and disorders.

In summary, the neurotrophin–Gal-3 axis represents a promising conceptual and therapeutic framework for understanding neuroinflammation (Figure 2). Further elucidation of this complex interplay may facilitate the development of novel biomarkers and more effective disease-modifying strategies in neurological and psychiatric disorders.

## Data Availability

No new data was created or analyzed in this study. Data sharing is not applicable.

## Abbreviations

The following abbreviations are used in this manuscript:

CNS	Central nervous system
Gal-3	Galectin-3
DCs	Dendritic cells
NK	Natural killer
NGF	Nerve growth factor
BDNF	Brain-derived neurotrophic factor
NT-3	Neurotrophin-3
NT-4/5	Neurotrophin-4/5
PI3K	Phosphoinositide 3-kinase
WT	Wild type
AD	Alzheimer’s disease
PD	Parkinson’s disease
MS	Multiple sclerosis
DAM	Disease-associated microglia
MGnD	Microglia neurodegenerative phenotype
CRP	C-reactive protein
CMS	Chronic mild stress
COX-2	Cyclooxygenase-2
NOS	Nitric oxide synthase
EOAD	Early-onset Alzheimer’s disease
LOAD	Late-onset Alzheimer’s disease
MCI	Mild cognitive impairment
MDD	Major depressive disorder
MMSE	Mini-Mental State Examination
mHTT	Mutant Huntingtin protein
EAE	Experimental autoimmune encephalomyelitis
CRDs	Conserved carbohydrate-recognition domains
FasL	Fas ligand
SCZ	Schizophrenia
HC	Healthy control
TRS	Treatment-refractory schizophrenia
mBDNF	Mature BDNF
BBB	Blood–brain barrier
ECT	Electroconvulsive therapy
